# Metabolic reprogramming in the spinal cord drives the transition to pain chronicity

**DOI:** 10.1016/j.celrep.2025.116261

**Published:** 2025-09-12

**Authors:** Alex Mabou Tagne, Yannick Fotio, Hye-Lim Lee, Kwang-Mook Jung, Jean Katz, Faizy Ahmed, Johnny Le, Richard Bazinet, Cholsoon Jang, Daniele Piomelli

**Affiliations:** 1 Department of Anatomy and Neurobiology, University of California, Irvine, Irvine, CA, USA; 2 Department of Biological Chemistry, University of California, Irvine, Irvine, CA, USA; 3 Department of Pharmaceutical Sciences, University of California, Irvine, Irvine, CA, USA; 4 Department of Nutritional Sciences, University of Toronto, Toronto, ON, Canada

## Abstract

Acute injuries can progress into painful states that endure long after healing. The mechanisms underlying this transition remain unclear, but metabolic adaptations to the bioenergy demands imposed by injury are plausible contributors. Here we show that peripheral injury activates AKT/mTORC1 in afferent segments of the mouse spinal cord, redirecting local core metabolism toward biomass production while simultaneously suppressing autophagy-mediated biomass reclamation. This metabolic shift supports neuroplasticity but creates a resource bottleneck that depletes critical spinal cord nutrients. Preventing this depletion with a modified diet normalizes biomass generation and autophagy and halts the transition to chronic pain. This effect, observed across multiple pain models, requires activation of the nutrient sensors, sirtuin-1 and AMPK, as well as restoration of autophagy. The findings identify metabolic reprogramming and consequent autophagy suppression as key drivers of the progression to pain chronicity and highlight nutritional and pharmacological interventions that could prevent this progression after surgery or other physical traumas.

## In brief

Mabou Tagne et al. show that peripheral injury reprograms spinal cord metabolism to support neuroplasticity but depletes nutrients and suppresses autophagy, promoting chronic pain. Dietary intervention restores metabolic balance and halts pain chronification, highlighting a possible strategy to block the transition to chronic pain.

## Graphical Abstract

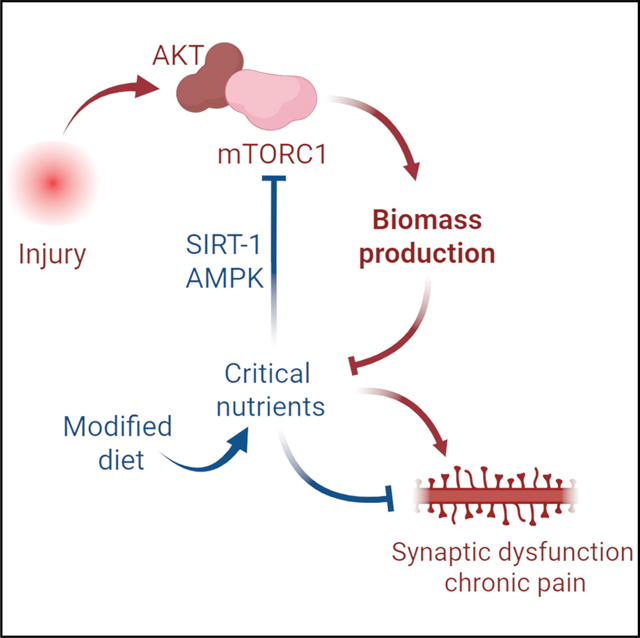

## INTRODUCTION

Acutely painful injuries can transition into intractable pain states that persist long after tissue healing.^1,2^ Invasive surgeries are a striking example of this progression, with 30%–50% of patients who undergo thoracotomy or mastectomy still reporting pain 1 year after an otherwise successful procedure.^3,4^ Similarly, up to 30% of people who experience accidental physical trauma go on to develop persistent neck or back pain.^5–7^ Stable neuroplastic modifications associated with central and peripheral sensitization underpin the emergence of chronic pain after injury,^8–10^ but the molecular determinants driving these changes are still elusive.^11,12^ Addressing this gap is crucial to identifying checkpoints in the transition to pain chronicity, which could be targeted by therapy.

Metabolic adaptations to the energetic challenges posed by injury are plausible contributors to chronic pain development. Synaptic transmission in the central nervous system (CNS) consumes a formidable amount of bioenergy.^13,14^ ATP, the product of these processes, powers action potentials, fuels transmitter release and vesicle recycling, and sustains synapse maintenance and remodeling. When an organ is damaged, bioenergy demands increase markedly as neurons and glia in afferent segments of the spinal cord must allocate their resources to two opposing tasks: supporting enhanced neural activity while simultaneously generating the biomass needed to establish central sensitization. Three modifications associated with the latter phenomenon—neuronal hyperexcitability,^8^ long-term heightening of synaptic strength,^8^ and accrued dendritic spine dynamics^15,16^—are, in fact, critically dependent on the production of new proteins and lipids.^17–21^ How spinal cord neurons and glia balance these conflicting demands is unclear. However, one possibility is that they reroute their metabolism toward aerobic glycolysis—a pathway that, while less energy-efficient than mitochondrial respiration, produces precursors for the biosynthesis of nucleotides, amino acids, and fatty acids.^22^

Animal studies support the possibility that somatic injury reprograms metabolism in the spinal cord. For instance, hind-paw administration of formalin in rats and chronic constriction injury (CCI) of the sciatic nerve in mice promote central sensitization and lasting pain through a mechanism that requires activation of the metabolic controller mammalian target of rapamycin complex 1 (mTORC1) and its upstream regulator AKT.^23–25^ Furthermore, hind-paw formalin injection in mice stimulates both the expression of glycolytic enzymes and the accumulation of glycolysis metabolites in lumbar spinal cord segments ipsilateral to the lesion, suggesting that glycolysis might be enhanced.^26^ Krebs cycle and oxidative phosphorylation components are concomitantly reduced, leading to decreased ATP levels. Importantly, this metabolic shift peaks 4 days after injury and coincides with a decline in the local concentrations of various amino acids, fatty acids, and the fatty acyl derivative palmitoylethanolamide (PEA). PEA is an endogenous agonist of peroxisome proliferator-activated receptor (PPAR)-α,^27^ a key transcriptional regulator of mitochondrial respiration.^28^ During this critical time window, but not before or after, PPAR-α activation halts the progression to pain chronicity.^26^ However, it remains unclear whether the injury-induced metabolic shift and nutrient deficit independently drive this progression.

To address this question, in the present study, we examined whether preserving normative levels of amino acids, fatty acids, and PEA in the spinal cord prevents the emergence of chronic pain following injury. We found that a modified diet (MD-1) that counters the injury-induced shortfall in nutrients and PEA stops both metabolic reprogramming and chronic pain development following hind-paw formalin injection in male and female mice. Similar effects were observed in three additional models of injury-induced pain—CCI,^29^ spared nerve injury (SNI),^30^ and surgical paw incision (SPI)^31^—but not in the complete Freund’s adjuvant (CFA) model of immune-induced pain. Importantly, a nutrient-matched diet isocaloric with MD-1 but lacking PEA or a diet enriched solely in PEA was not protective, indicating that the effects of MD-1 cannot be attributed to nutrients or PEA alone, but rather to a synergistic interaction between these two factors. In mechanistic investigations, we found that, in mice fed a standard diet, hind-paw damage activates AKT/mTORC1 signaling in the spinal cord, enhancing biomass production and suppressing autophagy. MD-1 normalizes both processes and stops the pain chronification by engaging the nutrient sensors, sirtuin-1 (SIRT1) and AMP-activated protein kinase (AMPK).^32,33^ These results identify critical injury-induced metabolic alterations in the spinal cord that drive the transition to pain chronicity. They also suggest that pharmacological and nutritional interventions that correct these alterations could be adopted in the clinic to prevent the transition to chronic pain following invasive surgery or other forms of physical trauma.

## RESULTS

### Peripheral injury promotes nutrient depletion in the spinal cord, and MD-1 averts it

Consistent with previous findings,^26^ hind-paw formalin injection induced metabolic reprogramming in ipsilateral lumbar (L4-L6) hemicords of male CD-1 mice, which peaked 4 days post-lesion (Figure S1).^26^ Compared to saline, formalin injection upregulated the transcription of genes involved in glycolysis, while genes associated with Krebs cycle and oxidative phosphorylation were generally, though not uniformly, downregulated (Figures S1A and S1B). Notable exceptions were succinate dehydrogenase (*Sdhb* and *Sdhd*) and ubiquinol-cytochrome c reductase (*Ucqrq*), whose transcription was elevated (Figure S1B), possibly due to their contribution to other consequences of injury, including suppression of autophagy and stimulation of apoptosis.^34,35^ Furthermore, levels of glycolytic metabolites were higher (Figure S1C) and Krebs cycle metabolites were lower relative to vehicle controls (Figure S1D). Prior work has shown that this metabolic shift is associated with a substantial reduction in the local concentrations of 12 amino acids, two monounsaturated fatty acids (oleic acid [18:1Δ^9^] and erucic acid [22:1Δ^13^]) and the endogenous PPAR-α agonist PEA.^27^ To determine whether this depletion independently contributes to pain chronification, we formulated an MD-1 enriched with the depleted substances (Table S1).

**Figure 1.**
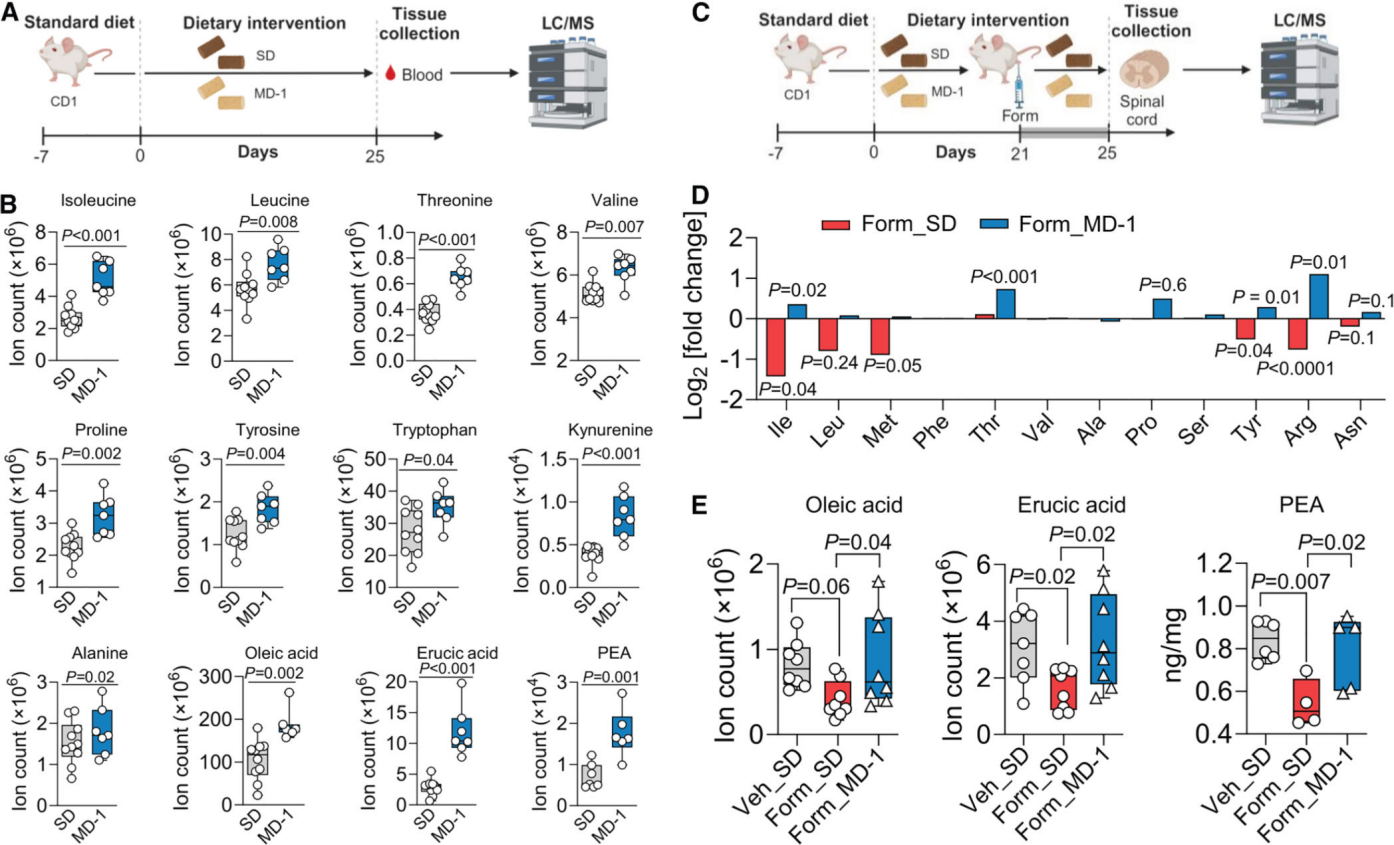
MD-1 is bioavailable and prevents injury-induced nutrient depletion in the spinal cord (A) Protocol: mice received SD or MD-1 for 25 days, and blood was collected for LC-MS analysis. (B) Serum concentrations (ion count) of MD-1 components in mice fed SD (gray boxes) or MD-1 (blue boxes). (C) Protocol: mice received SD or MD-1 for 21 days, followed by formalin (Form) or saline injection. Four days post-injection, ipsilateral L4-L6 spinal cord segments were collected for LC-MS analysis. (D) Effects of Form and MD-1 (log_2_ fold changes) on amino acid levels. Red bars: Form vs. vehicle in SD-1-fed mice; blue bars; MD-1 vs. SD feeding in Form-injected mice. (E) Oleic acid, erucic acid, and PEA content in vehicle-injected mice fed SD (gray boxes) and Form-injected mice fed SD (red boxes) or MD-1 (blue boxes). (B and D) Student’s t test, *n* = 6–10 per group; (E) One-way ANOVA and Šídák’s test, *n* = 7–8 per group.

Firstly, to assess the diet’s bioavailability, we fed male CD-1 mice MD-1 for 25 days and analyzed blood samples using liquid chromatography/mass spectrometry (LC-MS) (Figure 1A). Compared to mice on a standard diet (SD), mice fed MD-1 showed significantly higher serum concentrations of most supplemented compounds (Figure 1B). The concentrations of serine and phenylalanine remained unchanged, likely due to biotransformation, as indicated by higher levels of their catabolites, glycine (from serine) and phenylpyruvate (from phenylalanine) (Figures S2A and S2B). Elevated serum concentrations of various tryptophan and tyrosine catabolites confirmed that MD-1 components were integrated into core metabolism (Figures 1B, S2C, and S2D). The absence of detectable cysteine, along with unaltered cysteine catabolites, methionine, and methionine catabolite cystathionine (Figures S2E and S2F), may reflect incorporation into proteins or conversion into compounds not targeted in our analysis. Other serum metabolites influenced by MD-1 are listed in Table S2.

We next examined whether MD-1 prevents the injury-induced nutrient deficit in ipsilateral lumbar hemicord tissue. CD-1 mice were fed MD-1 or SD for 21 days before receiving injections of formalin (1%, 20 μL) or saline. This model is commonly used to study acute pain^36^ but also induces key aspects of severe chronic pain in humans,^26^ including bilateral hypersensitivity,^37,38^ heightened anxiety,^39^ cognitive impairments,^40^ and structural CNS alterations.^41^ Four days post-formalin, we harvested ipsilateral lumbar hemicords and quantified metabolites by LC-MS (Figure 1C). As previously observed in C57Bl6 mice,^26^ formalin-treated, SD-fed mice exhibited lower levels of various amino acids, oleic acid, erucic acid, and PEA compared to vehicle-injected controls (Figures 1D and 1E). No such decline was observed, however, in formalin-treated, MD-1-fed mice, where levels of these substances were either stable or slightly elevated compared to uninjured mice (Figures 1D and 1E). In the latter, MD-1 increased concentrations of PEA and 4 of the 12 amino acids supplemented by the diet (Figure S3A). Other metabolomic changes elicited by MD-1 in ipsilateral lumbar hemicords of uninjured mice are detailed in Table S3. MD-1’s ability to avert spinal nutrient depletion encouraged us to utilize this diet to investigate the relationship between injury-induced metabolic alterations and chronic pain development.

### MD-1 prevents injury-induced metabolic reprogramming in the spinal cord

**Figure 2.**
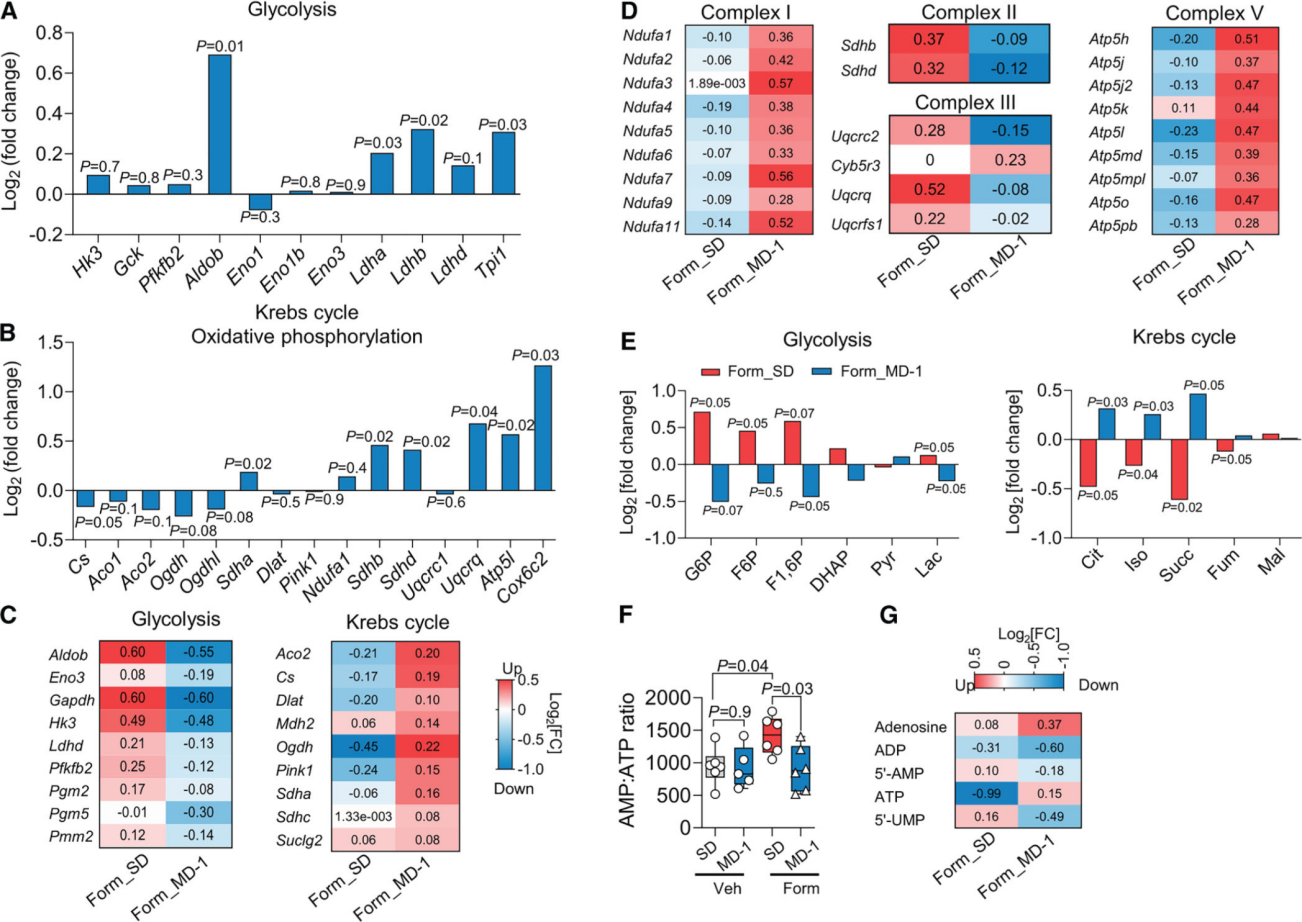
MD-1 prevents injury-induced metabolic reprogramming in the spinal cord (A and B) Effects of MD-1 on the transcription of genes related to (A) glycolysis and (B) Krebs cycle and oxidative phosphorylation. Log_2_ fold changes in MD-1-fed vs. SD-fed vehicle (Veh)-injected mice. (C and D) Effects of formalin and MD-1 (log_2_ fold changes) on the transcription of genes related to (C) glycolysis and Krebs cycle, and (D) oxidative phosphorylation. Form_SD: formalin (Form) vs. Veh injection in SD-1-fed mice; Form_ MD-1: MD-1 vs. SD feeding in formalin-injected mice. (E) Effects of formalin and MD-1 (log_2_ fold changes) on the concentrations of glycolysis (left) and Krebs cycle (right) metabolites. Form_SD: Form vs. Veh injection in SD-1-fed mice; Form_ MD-1: MD-1 vs. SD feeding in Form-injected mice. Abbreviations: G6P, glucose-6-phosphate; F6P, fructose-6-phosphate; F1,6P, fructose-1–6-bisphosphate; DHAP, dihydroxyacetone phosphate; Pyr, pyruvate; Lac, lactate; Cit, citrate; Iso, isocitrate, Succ, succinate; Fum, fumarate; Mal, malate. (F) AMP/ATP ratio in Veh- or Form-injected mice fed SD or MD-1. (G) Effects of Form and MD-1 (log_2_ fold changes) on nucleoside and nucleotide concentrations. Form_SD: Form vs. Veh injection in SD-1-fed mice; Form_ MD-1: MD-1 vs. SD feeding in Form-injected mice. (A, B, and E) Multiple unpaired t test, *n* = 6–10 per group; (F) One-way ANOVA and Šídák’s test, *n* = 5–6 per group.

Figure 2 illustrates the effects of MD-1, alone or in combination with formalin, on the expression of genes associated with energy metabolism in ipsilateral lumbar hemicords, as assessed by bulk RNA sequencing (RNA-seq). In uninjured mice, MD-1 enhanced the transcription of critical glycolytic genes (Figure 2A). The diet also increased the expression of oxidative phosphorylation components, such as ATP synthase subunit g (*Atp5l*), cytochrome c oxidase subunit 6C2 (*Cox6c2*), *Sdh (a*, *b*, and *d)*, and *Ucqrq1* (Figure 2B). Additionally, MD-1 induced a downward trend in the transcription of Krebs cycle-related genes, with citrate synthase (*Cs*) showing borderline statistical significance (*p* = 0.05) (Figure 2B). However, these transcriptional modifications, along with others reported in Table S4, were not accompanied by detectable changes in the levels of glycolysis or Krebs cycle metabolites (Figures S3B and S3C). In contrast, MD-1 effectively counteracted all molecular changes caused by formalin injection in ipsilateral lumbar hemicords, blocking the upregulation of glycolytic genes and the downregulation of genes involved in Krebs cycle and oxidative phosphorylation (Figures 2C and 2D; see Table S4 for statistical analyses) as well as the shifts in glycolysis and Krebs cycle metabolites (Figure 2E). Notably, MD-1 preserved normative ATP levels in formalin-treated mice without altering them in controls (Figure 2F). Changes in other purines were also blocked by MD-1 (Figure 2G). Thus, MD-1 has a limited impact on bioenergy production in uninjured mice but averts metabolic reprogramming after injury.

The serine/threonine kinase AKT and its downstream target mTORC1 are pivotal regulators of energy metabolism.^42,43^ They are also required for the induction of central sensitization and chronic pain following formalin injection in rats or sciatic nerve injury in mice.^23–25^ In line with this role, formalin administration in SD-fed mice increased AKT and mTOR phosphorylation (activation) in ipsilateral lumbar hemicords (Figures S4A–S4C). This coincided with enhanced transcription of phosphoinositide-3-kinase catalytic subunit-γ (*Pik3cg*) (Figure S4D), which facilitates AKT recruitment,^44^ and reduced transcription of regulated in development and DNA damage 1 (*Redd1*), an endogenous AKT/mTORC1 inhibitor,^45^ as shown by bulk RNA-seq (Figure S4E). Additionally, PPAR-α (*Ppara*) and peroxisome proliferator-activated receptor-γ coactivator-1α ( *Ppargc1a*)—key controllers of mitochondrial respiration^46^ that are repressed by mTORC1^47^—were downregulated in formalin-treated mice, compared to controls (Figures S4F and S4G), whereas PPAR-γ (*Pparg*) and PPAR-δ (*Ppard*) remained unchanged (Figure S4H). Notably, MD-1 prevented these alterations (Figures S6A–S6H) and decreased baseline AKT phosphorylation in control mice (Figures S4A and S4B).

To determine whether AKT and mTORC1 are involved in chronic pain development, we treated SD-fed mice with the AKT inhibitor MK-2206 (240 mg/kg, intraperitoneal, i.p.)^48^ or the mTORC1/2 inhibitor Torin-1 (20 mg/kg, i.p.)^49^ once daily on days 2–4 post-formalin (Figure S5A). Both inhibitors halted the development of bilateral hypersensitivity (Figures S5B and S5C), confirming that AKT/mTORC1 signaling is necessary to establish central sensitization and chronic pain.^23–25^ These data, along with the known anabolic functions of mTORC1,^42,43^ led us to hypothesize that injury-induced mTORC1 activation drives the progression to pain chronicity by affecting biomass production and autophagy.

### Injury enhances biomass production and suppresses autophagy in the spinal cord, and MD-1 prevents these effects

**Figure 3.**
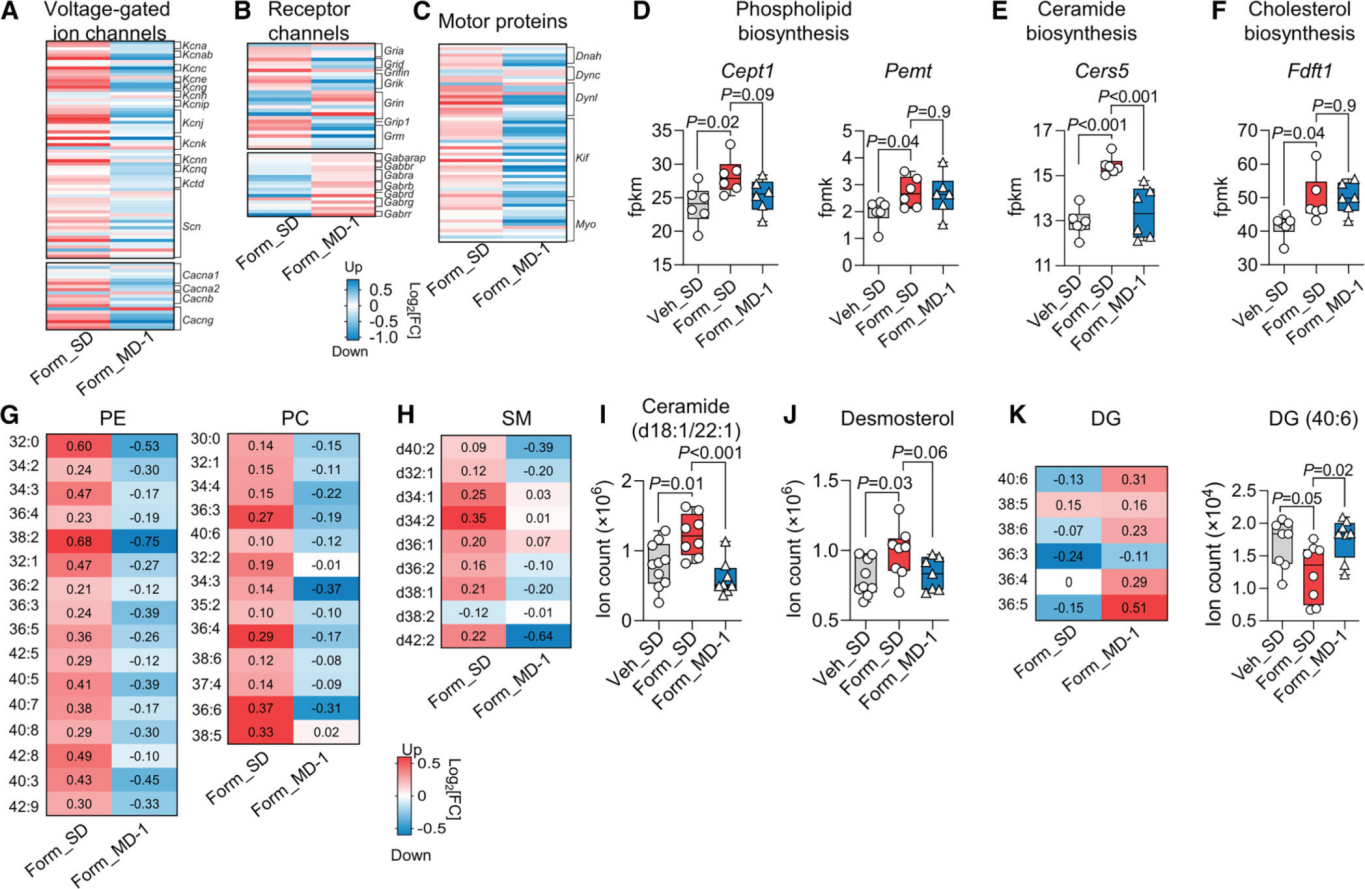
MD-1 prevents injury-induced biomass generation in the spinal cord (A–C) Effects of formalin and MD-1 (log_2_ fold changes) on the transcription of genes related to (A) voltage-gated ion channels, (B) receptor channels, and (C) motor proteins. Form_SD: formalin vs. vehicle injection in SD-1-fed mice; and Form_MD-1: MD-1 vs. SD feeding in formalin-injected mice. (D–F) Effects of formalin and MD-1 on the transcription (fpkm) of genes encoding biosynthetic enzymes for (D) glycerophospholipids (choline/ethanolamine phosphotransferase 1, *Cept1*), (E) ceramide (ceramide synthase 5, *Cers5*), and (F) cholesterol (squalene synthase, *Fdft1*). Veh_SD: vehicle-injected mice fed SD; Form_SD: formalin-injected mice fed SD; Form_MD-1: formalin-injected mice fed MD-1. (G and H) Effects of formalin and MD-1 (log_2_ fold changes) on the levels of (G) PE and PC, and (H) sphingomyelins (SM). Form_SD: formalin vs. vehicle injection in SD-1-fed mice; and Form_MD-1: MD-1 vs. SD feeding in formalin-injected mice. (I and J) Effects of formalin and MD-1 on levels (ion count) of (I) ceramide (Δ18:1/22:1) and (J) desmosterol. Veh_SD: vehicle-injected mice fed SD; Form_SD: formalin-injected mice fed SD; and Form_MD-1: formalin-injected mice fed MD-1. (K) Effects of formalin and MD-1 on DG levels (ion count). Form_SD: formalin vs. vehicle injection in SD-1-fed mice; Form_ MD-1: MD-1 vs. SD feeding in formalin-injected mice. (D and I–K) One-way ANOVA and Šídák’s test; *n* = 6–8 per group.

The neuroplastic adaptations that underlie central sensitization depend on the synthesis of synaptic proteins and lipids.^17–21^ Accordingly, bulk RNA-seq studies showed that the transcription of numerous genes involved in the production of neuroglial biomass was enhanced in ipsilateral lumbar hemicords of SD-fed, formalin-treated mice relative to uninjured controls (Figure 3; see Table S5 for statistical analyses). For example, there was an upregulation of genes encoding voltage-gated ion channels, receptor channels, and motor proteins (Figures 3A–3C) along with genes required for the synthesis of glycerophospholipids, sphingolipids, and cholesterol (Figures 3D–3F). These transcriptional modifications aligned with accrued levels of glycerophospholipids (Figure 3G), sphingolipids (Figures 3H and 3I), and desmosterol (Figure 3J), as determined by metabolomics. Diacylglycerols were concomitantly decreased (Figure 3K), likely due to their use in phospholipid biosynthesis.^50^ In contrast, MD-1-fed mice were strikingly resilient to the anabolic stimulation evoked by injury. In formalin-treated mice receiving MD-1, the diet prevented the upregulation of genes encoding ion channels, motor proteins, and lipid synthetic enzymes (Figures 3A–3F), and stabilized tissue concentrations of all lipid classes (Figures 3G–3K). The results indicate that injury stimulates the production of neuroplasticity-associated proteins and lipids in the spinal cord, likely under the control of mTORC1,^42,43^ and MD-1 blocks this response.

**Figure 4.**
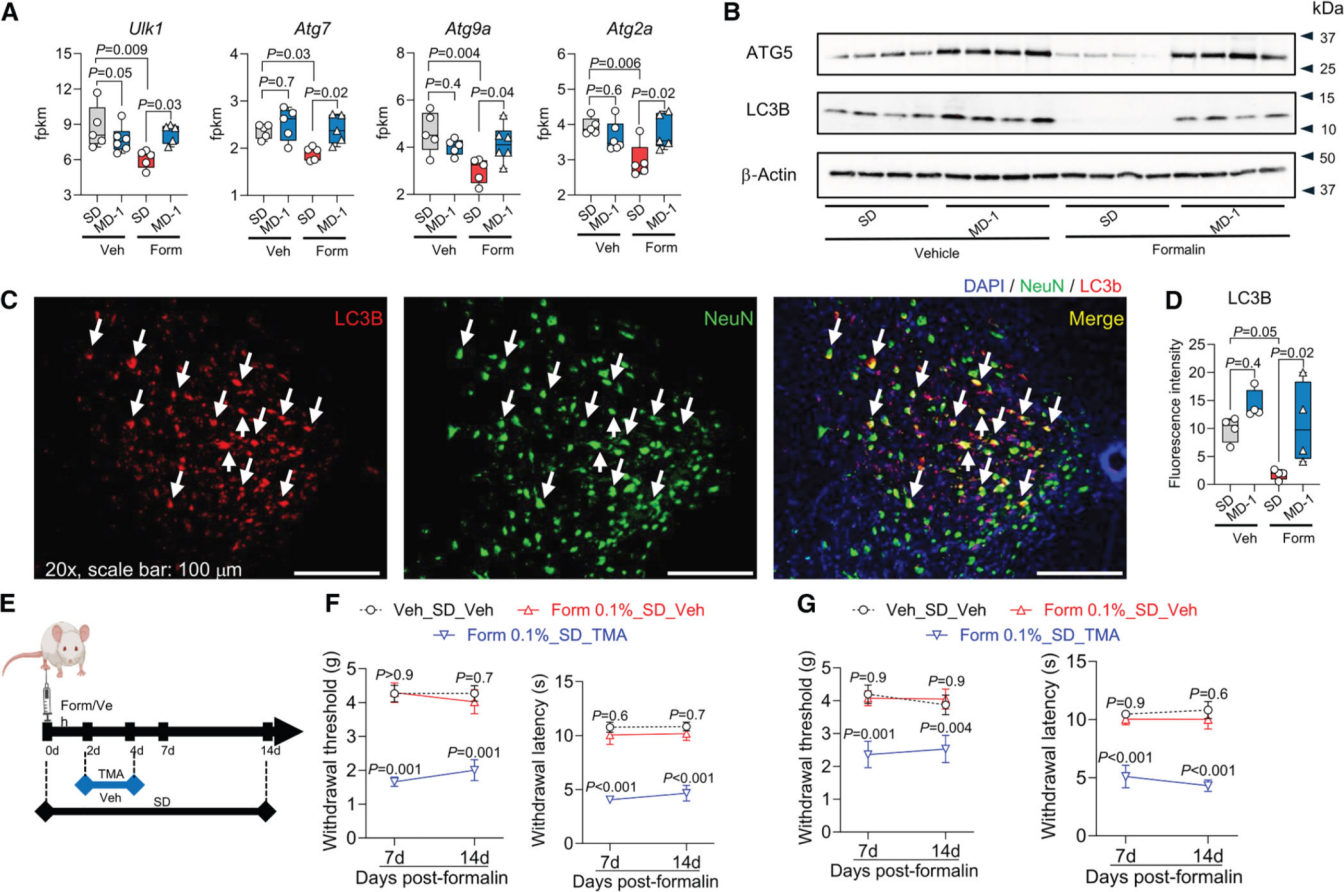
Injury-induced autophagy suppression in the spinal cord facilitates the transition to chronic pain, and MD-1 prevents autophagy suppression (A) Transcription (fpkm) of key autophagy regulators in vehicle (Veh)- or formalin (Form)-injected mice fed SD or MD-1. (B) Representative western blot images showing ATG5 and LC3B content in Veh- and Form-injected mice fed either SD or MD-1. β-actin is the loading control. (C) Representative immunofluorescent images for LC3B (red) and neuronal marker NeuN (green) in the L4-L6 spinal cord of Form-injected mice fed MD-1. Nuclei are stained with DAPI. Arrows indicate LC3B and NeuN colocalization. Magnification: 20×. Scale bars, 100 μm. (D) Quantification of LC3B immunofluorescence in the L4-L6 spinal cord of Veh- or Form-injected mice fed SD or MD-1. (E) Protocol: Autophagy inhibitor TMA (30 mg/kg, i.p.) or its Veh was administered to SD-fed mice on days 2–4 after Form injection. (F and G) Effects of TMA on (F) contralateral or (G) ipsilateral hypersensitivity to mechanical (left) and thermal (right) stimuli. Open circles: Veh/SD/Veh; red triangles: Form/SD/Veh; blue triangles: Form/MD-1/Veh; and open squares, Form/MD-1/TMA. (A, B, D, F, and G) One- or two-way ANOVA followed by Dunnett or Šídák’s test. *p < 0.05, **p < 0.01, and ***p < 0.001 compared to formalin-SD (*n* = 7–10).

In addition to stimulating biomass production, formalin injection in SD-fed mice reduced the transcription of autophagy regulators unc-51-like autophagy-activating kinase 1 (*Ulk1*) and autophagy-related genes (*Atg*) 7, *Atg9a*, and *Atg2a* in ipsilateral lumbar hemicords (Figure 4A). Protein levels of ATG5 and micro-tubule-associated protein 1 light chain 3b (LC3B) were also lower, compared to uninjured mice, as assessed by both immunoblot (Figures 4B, S5D, and S5E) and immunofluorescence analyses, which also identified neurons as one of the cell types involved in this response (Figures 4C, 4D, and S5F). MD-1 prevented the effects of injury, normalizing *Ulk1*, *Atg7*, *Atg9a*, and *Atg2a* transcription and restoring LC3B and ATG5 protein levels (Figures 4A–4D and S5D–S5F). To determine whether autophagy suppression contributes to pain chronification, we administered the autophagy inhibitor 3-methyl adenine (TMA, 30 mg/kg, i.p.) to SD-fed mice treated with a formalin dose (0.1%, 20 μL) that is insufficient to induce chronic pain^26^ (Figure 4E). TMA administration on days 2–4 after low-dose formalin was followed by robust and sustained bilateral hypersensitivity (Figures 4F and 4G), indicating that autophagy inhibition enables minor injuries to cause enduring pain. In contrast, injection of low-dose formalin without TMA or saline plus TMA had no such effect (Figures 4F and 4G).

Autophagy suppression promotes apoptosis.^51^ Accordingly, formalin injection in SD-fed mice increased the transcription of proapoptotic genes in L4-L6 spinal cord while concurrently downregulating survival genes such as *Bcl2* and *Mcl1* (Figure S6A). Immunofluorescence analyses revealed elevated levels of activated caspase-3 immunoreactivity in neurons and other cells of the lumbar spinal cord (Figure S6B). MD-1 did not influence the expression of apoptosis-related genes in uninjured mice, but effectively blocked the changes evoked by formalin (Figures S6A and S6B). To determine whether apoptosis contributes to pain chronification, we administered the pan-caspase inhibitor emricasan^52^ (3 mg/kg, i.p.) to SD-fed mice treated with 1% formalin. Emricasan administration on days 2–4 post-formalin normalized cleaved caspase-3 levels in the lumbar spinal cord (Figures S6C and S6D), indicating that it effectively halted apoptosis. Notably, however, emricasan failed to prevent the transition to persistent hypersensitivity (Figure S7), indicating that apoptosis stimulation—unlike autophagy suppression—is not involved in pain chronification.

Collectively, these findings provide two key insights: (1) peripheral injury suppresses autophagy in spinal cord neurons, and this suppression plays a critical role in the progression to pain chronicity; and (2) autophagy suppression is accompanied by an increase in neuronal apoptosis, which, however, does not contribute to pain chronification.

### Injury alters the systemic metabolome, and MD-1 prevents this effect

Spinal cord changes were accompanied by marked alterations in the circulating metabolome (Figure S8A; Table S6). On day 4 post-formalin, serum concentrations of corticosterone and aldosterone were elevated in SD-fed mice, indicating sustained activation of the hypothalamic-pituitary-adrenal axis and reninangiotensin system (Figure S8B). Additionally, levels of 3-methylglutarylcarnitine, a potential marker of mitochondrial dysfunction,^53^ and taurine, a substance with anti-apoptotic properties,^54^ were increased (Figure S8C). Serum accumulation of phosphatidylcholine (PC) and sphingomyelin (SM) mirrored similar alterations in the spinal cord (Figure S8D). Also consistent with spinal cord findings, various diacylglycerols and long-chain fatty acids—erucic, nervonic (24:1Δ^15^), and docosatrienoic (22:3Δ^13,16,19,^)—were lower in formalin-treated mice (Figure S8E). MD-1 countered these trends, normalizing serum corticosterone, aldosterone, 3-methylglutarylcarnitine, and taurine (Figures S8B and S8C), while resulting in lower PC and SM levels (Figure S8D), and replenishing diacylglycerols and fatty acids (Figure S8E). Importantly, the effects of MD-1 did not reflect modifications in the intestinal microbiome: fecal samples analysis showed that, compared to SD, a 25-day exposure to MD-1 did not influence the Shannon diversity index (Figure S9A) or the relative abundance of intestinal bacterial genera (Figures S9B and S9C). Thus, chronic pain development is associated with profound changes in the systemic metabolome, which partially reflect those in ipsilateral spinal hemicords. These changes are unaffected by gut microbiome composition and are prevented by MD-1.

### MD-1 prevents the transition to chronic pain following peripheral injury

**Figure 5.**
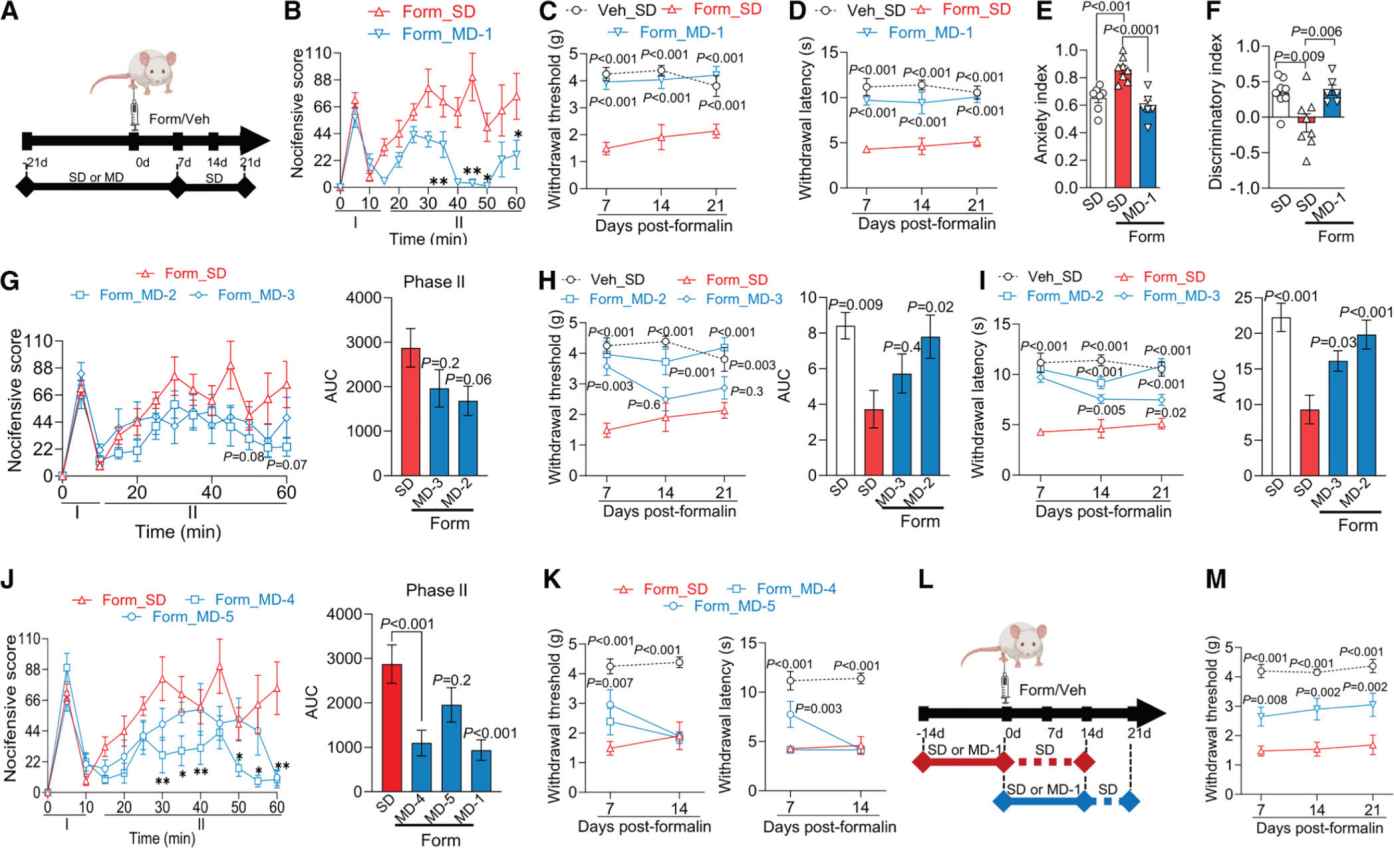
MD-1 prevents the progression to pain chronicity in a dose-, composition-, and time-dependent manner (A) Mice received SD or a modified diet (MD-1, MD-2, MD-3, MD-4, or MD-5) for 21 days before formalin (Form) or saline injection. Access to the modified diets was extended for 1 week after injection, and pain behaviors were monitored for the following 3 weeks. (B) Time-course (min) of the acute nocifensive response to Form in mice fed SD (red triangles) or MD-1 (blue triangles). The response has two temporally distinct phases (I and II). (C and D) Time-course (days) of contralateral hypersensitivity to (C) mechanical and (D) thermal stimuli in Form-injected mice fed SD (red triangles) or MD-1 (blue triangles). Open circles indicate vehicle (Veh)-injected mice fed SD. (E) Anxiety-like behavior (elevated plus-maze) in Vehi-injected mice fed SD (open bar) and Form-injected mice fed SD (red bar) or MD-1 (blue bar). (F) Long-term memory (24-h novel-object recognition) in Veh-injected mice fed SD (open bar) and Form-injected mice fed SD (red bar) or MD-1 (blue bar). (G) Time-course (min) of the nocifensive response to Form in mice fed SD (red triangles), MD-2 (half-dose MD-1, blue squares), or MD-3 (quarter-dose MD-1, blue diamonds). Right panel: quantification (area under the curve, AUC) for phase II of the formalin response in mice fed SD (red bar), MD-2, or MD-3 (blue bars). (H and I) Time-course (days) of contralateral hypersensitivity to (H) mechanical and (I) thermal stimuli in Form-injected mice fed SD (red triangles), MD-2 (blue squares), or MD-3 (blue diamonds). Right panels: AUC quantification for the interval between 7 and 21 days. (J) Time-course (min) of the nocifensive response to Form in mice fed SD (red triangles), MD-4 (blue squares), or MD-5 (blue circles). Right panel: AUC values for phase II of the Form response in mice fed SD (red bar), MD-4, MD-5, or MD-1 (blue bars). (K) Time-course (days) of contralateral hypersensitivity to (left) mechanical and (right) thermal stimuli in Form-injected mice fed SD (red triangles), MD-4 (blue squares), or MD-5 (blue circles). (L) Protocol for pre- and post-injury MD-1 administration. (M) Time-course (days) of contralateral mechanical hypersensitivity in Form-injected mice fed SD (red triangles) or MD-1 (blue triangles) post-injury. Open circles indicate Veh-injected mice fed SD. (B–K and M) One- or two-way ANOVA followed by Dunnett multiple comparisons test. *p < 0.05, **p < 0.01, and ***p < 0.001 (Form vs. Veh).

The findings thus far suggest that AKT/mTORC1 activation in the spinal cord stimulates biomass production and autophagy suppression, promoting a localized nutrient deficit that facilitates chronic pain development. To further test this hypothesis, we examined whether MD-1 interrupts this transition. We injected formalin or saline in male SD- or MD-1-fed mice and monitored them for the subsequent 3 weeks, with access to MD-1 extended for 1 week after injection (Figure 5A). As expected,^36^ in the SD-fed group, formalin elicited a nocifensive response (Figure 5B) accompanied by paw inflammation (Figure S10A). This acute reaction was followed by an enduring pathological state that exhibited three hallmarks of severe chronic pain in humans^26^: bilateral hypersensitivity to mechanical and thermal stimuli (contralateral: Figures 5C and 5D; ipsilateral; Figure S10B),^37,38^ heightened anxiety-like behavior^39^ (Figures 5E and S10C), and long-term memory deficits (Figure 5F).^40^ MD-1 reduced the second, but not the first, phase of the formalin response (Figure 5B), attenuated edema (Figure S10A), and, most crucially, stopped all behavioral signs of chronic pain (Figures 5C–5F, S10B, and S10C). Comparable effects were seen in female mice (Figure S10D–S10H), though MD-1 did not normalize anxiety-like behavior in females (Figure S10I). Due to its slight caloric enrichment (SD = 3.2 kcal/g vs. MD-1 = 3.7 kcal/g, Table S1), male mice fed MD-1 showed a small but statistically detectable difference in body-weight gain compared to SD-fed controls (Figure S10J). However, by covariance analysis, we found that this difference did not affect pain outcomes (Table S7). Additionally, corroborating our behavioral results, bulk RNA-seq analyses showed that the transcription of several proinflammatory genes—including interleukin 1β (*Il1b*), interleukin 6 (*Il6*), and tumor-necrosis factor-α (*Tnf*)^55,56^—was enhanced in ipsilateral lumbar hemicords of SD-fed formalin-treated mice, compared to vehicle-injected controls, but not in mice receiving MD-1 (Figure S11A). Expression of transforming growth factor β3 (*Tgfb3*), which may have tissue-reparative functions,^57^ was lower in injured mice, an effect also blocked by MD-1 (Figure S11B).

We next assessed whether dosage, chemical composition, or timing of administration influences MD-1’s efficacy. Firstly, we compared MD-1 with MD-2 and MD-3, two diets containing the same ingredients as MD-1 but at half and one-quarter the dosages, respectively (Table S1). We fed mice SD, MD-1, MD-2, or MD-3 for 21 days, followed by formalin or saline injection, with access to the diets extended for 1 week post-injection, and then tracked pain-related behaviors over the following 3 weeks. Unlike MD-1, MD-2 and MD-3 had no effect on formalin-induced acute nociception or inflammation (Figures 5G and S11C). Additionally, MD-2 blocked the development of persistent hypersensitivity, whereas MD-3 produced only transient and partial protection (contralateral: Figures 5H and 5I; ipsilateral: Figure S11D). The findings suggest that MD-1 prevents chronic pain development in a dose-dependent manner, underscoring the specificity of its action.

We then investigated whether MD-1’s effects could be attributed to its nutrient content, the presence of PEA, or their combination. To address this, we tested two additional diets: MD-4, which matches MD-1 in nutrient composition and energy density but does not contain PEA, and MD-5, an SD supplemented exclusively with PEA (Table S1). MD-4 attenuated the second phase of the acute nocifensive response to formalin (Figure 5J) but did not prevent edema formation or persistent hypersensitivity (contralateral: Figure 5K; ipsilateral: Figures S12A and S12B). In contrast, MD-5 did not affect formalin-induced nociception (Figure 5J) but reduced edema (Figure S12C) and delayed, without stopping, hypersensitivity development (contralateral: Figure 5K; ipsilateral: Figure S12B). Interestingly, although neither MD-4 nor MD-5 affected the emergence of hypersensitivity, both diets had distinct effects on the emotional and cognitive sequelae of injury: MD-4 improved memory but failed to alleviate anxiety-like behavior, whereas MD-5 reduced anxiety-like behavior but had only a borderline effect (*p* = 0.05) on memory (Figures S12D and S12E). The results suggest that MD-1’s combination of amino acids, fatty acids, and PEA is essential to provide comprehensive protection against pain chronification after injury.

Finally, we investigated whether the timing of MD-1 exposure—either prior to or following injury—would influence the diet’s efficacy. Mice were either fed MD-1 for 2 weeks leading up to the day of formalin injection, at which point they were switched to an SD, or they began the MD-1 diet on the day of the injection, which was continued for 2 weeks before the mice were returned to SD feeding (Figure 5L). Post-formalin MD-1 exposure partially prevented hypersensitivity (Figures 5M and S13A–S13C) and reduced the memory deficit (Figure S13D) but had no effect on anxiety-like behavior (Figure S13E). On the other hand, limiting the mice to pre-formalin MD-1 exposure caused only a modest decrease in ipsilateral mechanical hypersensitivity (Figure S13F) without altering other pain-related responses (Figure S13G–S13K). The findings indicate that MD-1 administration in the weeks after—but not before—formalin injection provides some protection, reaffirming the centrality of this time window in chronic pain consolidation.^26^ Full efficacy is observed, however, only when MD-1 is given both pre- and post-injury, suggesting that preemptive exposure increases systemic stores of depletion-prone nutrients.

### MD-1 prevents pain chronification by activating SIRT1 and AMPK and restoring autophagy

**Figure 6.**
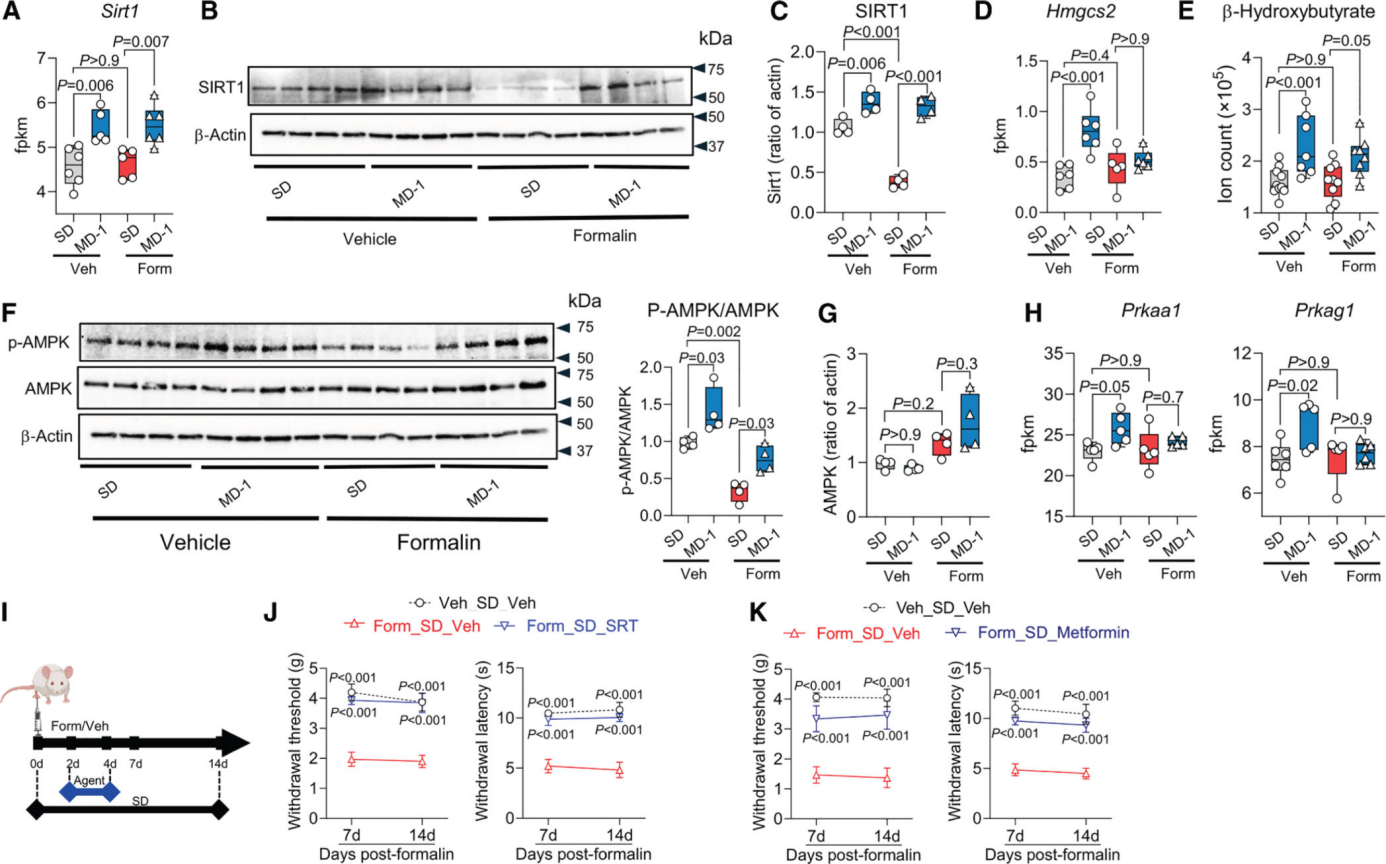
SIRT1 and AMPK mediate the protective effects of MD-1 (A) *Sirt1* gene transcription (fpkm) in the L4-L6 spinal cord of vehicle (Veh)- or formalin (Form)-injected mice fed SD or MD-1. (B) Representative western blot images showing SIRT1 protein content in Veh- or Form-injected mice fed SD or MD-1. β-actin is the loading control. (C) SIRT1 protein quantification (SIRT1/β-actin) in Vehe- or Form-injected mice fed SD or MD-1. (D) *Hmgcs2 t*ranscription in Veh- or Form-injected mice fed SD or MD-1. (E) β-hydroxybutyrate levels in Veh- or Form-injected mice fed SD or MD-1. (F) Representative western blot images showing phosphorylated AMPK (*p*-AMPK) and total AMPK levels. Right: *p*-AMPK quantification (*p*-AMPK/AMPK). (G) AMPK protein quantification (AMPK/β-actin) in Veh- or Form-injected mice fed SD or MD-1. (H) Transcription of AMPK subunits, *Prkaa1* and *Prkag1*, in Veh- or Form-injected mice fed SD or MD-1. (I) Protocol: SIRT1 activator SRT-2104 (100 mg/kg, IP), AMPK activator metformin (200 mg/kg, IP), or their Veh was administered to SD-fed mice on days 2–4 after Form injection. (J and K) Effects of (J) SRT-2104 and (K) metformin on contralateral hypersensitivity to mechanical (left) and thermal (right) stimuli. (A, C–E, G, H, J, and K) One- or two-way ANOVA with Dunnett or Šídák’s multiple comparisons test. *p < 0.05, **p < 0.01, and ***p < 0.001 compared to Form/SD (*n* = 4–8).

SIRT1 stimulates autophagy via both direct and indirect mechanisms,^58^ prompting us to test whether this nutrient sensor^33,59^ could contribute to the protective effects of MD-1. Transcriptomic and immunoblot experiments confirmed this possibility, showing that expression of SIRT1, but not other sirtuin family members, was elevated in ipsilateral lumbar hemicords of uninjured mice receiving MD-1 compared to SD-fed controls (Figures 6A–6C and S14A). Additionally, formalin administration markedly reduced SIRT1 protein levels, but not *Sirt1* transcription (Figures 6A–6C), suggesting that injury may stimulate SIRT1 proteolysis.^60^ In uninjured mice, MD-1 also enhanced transcription of the ketogenic enzyme, hydroxymethylglutaryl-CoA synthase-2 (*Hmgcs2*) (Figure 6D), which is indirectly regulated by SIRT1.^61^ This transcriptional activation correlated with accrued β-hydroxybutyrate levels (Figure 6E). *Hmgcs2* transcription and ketone body production were not affected by formalin treatment (Figures 6D and 6E).

We examined whether AMPK, another nutrient sensor,^62^ also contributes to MD-1’s actions. Three observations supported this possibility (Figure 6F): (1) MD-1 increased AMPK phosphorylation in uninjured mice; (2) formalin injection decreased AMPK phosphorylation; and (3) MD-1 negated formalin’s effect. AMPK protein levels were not altered by either MD-1 or formalin (Figure 6G). The transcription of two AMPK subunits (*Prkaa1* and *Prkag1*) was stimulated by MD-1 but was not affected by formalin (Figure 6H), while transcription of other subunits (*Prkaa2* and *Prkab2*) was reduced by formalin and normalized by MD-1 (Figure S14B).

These changes suggest that SIRT1 and AMPK are involved in the protective effects of MD-1. To assess SIRT1’s role, we administered the SIRT1 activator SRT-2104 (100 mg/kg, i.p.) on days 2–4 after injection of 1% formalin (Figure 6I). SRT-2104 blocked the development of hypersensitivity (Figures 6J, S15A, and S15B) but failed to prevent paw edema (Figure S15C). Next, we examined the effects of the SIRT1 inhibitor EX-527 (20 or 50 mg/kg, i.p.) administered on days 2–4 after injection of 0.1% formalin, which does not cause persistent hypersensitivity.^25^ Treatment with 50 mg/kg EX-527 was followed by robust and sustained bilateral hypersensitivity (Figures S15D–S15G), indicating that SIRT1 inhibition facilitates pain chronification. A weaker response was observed at the 20 mg/kg dose (Figures S15D–S15G).

To evaluate AMPK’s contribution to the protective effects of MD-1, we administered the AMPK activator metformin (200 mg/kg, i.p.) to SD-fed mice on days 2–4 after injection of 1% formalin (Figure 6I). Unlike SRT-2104, metformin prevented the development of contralateral but not ipsilateral hypersensitivity (Figures 6K, S16A, and S16B). Like SRT-2104, however, metformin did not affect formalin-induced paw edema (Figure S16C).

Given that both SIRT1 and AMPK promote autophagy,^33,59,62^ we next examined whether inhibiting this process would negate the protective effects of MD-1. We treated SD- or MD-1-fed mice with either of two autophagy inhibitors—TMA (30 mg/kg, i.p.) or bafilomycin A1 (1 mg/kg, IP)—on days 2–4 post-formalin and tracked pain-related behaviors over the subsequent 2 weeks. Both inhibitors abolished MD-1’s effects, demonstrating their dependence on autophagy activation (Figure S17). These results indicate that MD-1 prevents the transition to pain chronicity through a mechanism that involves the activation of SIRT1 and AMPK and the consequent restoration of autophagy.

### MD-1 protects mice from post-surgical acute and chronic pain

**Figure 7.**
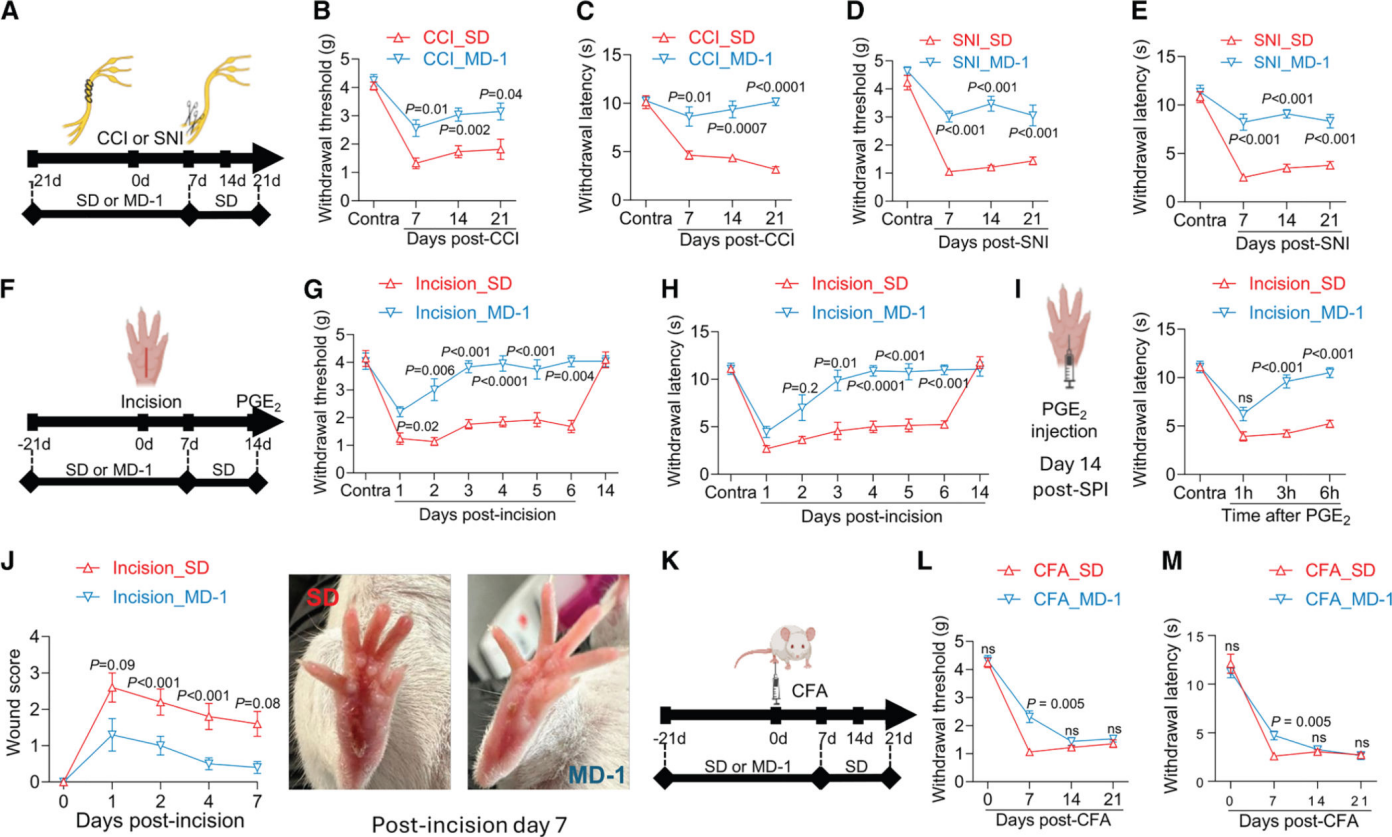
MD-1 prevents the development of acute and chronic pain after surgery (A) CCI and SNI models: mice were fed SD or MD-1 for 21 days before surgeries. MD-1 access was extended for 1 week post-surgery, and nocifensive behavior was monitored for the following 2 weeks. (B and C) Time-course of CCI-induced ipsilateral hypersensitivity (B, mechanical; and C, thermal) in mice fed SD (red triangles) or MD-1 (blue triangles). (D and E) Time-course of SNI-induced ipsilateral hypersensitivity (D, mechanical and E, thermal) in mice fed SD (red triangles) or MD-1 (blue triangles). (F) SPI model: mice were fed SD or MD-1 for 21 days before SPI. MD-1 access was extended for 1 week after SPI, and behavior was monitored for 2 more weeks. On day 14 post-SPI, the mice were given PGE_2_ (100 ng/20 mL, SC) in the lesioned paw, and nocifensive behavior was monitored for 6 h. (G and H) Time-course of SPI-induced ipsilateral hypersensitivity (G, mechanical; and H, thermal) in mice fed SD (red triangles) or MD-1 (blue triangles). (I) PGE_2_-induced ipsilateral thermal hypersensitivity in mice fed SD or MD-1. (J) Time-course (days) of first-intention wound healing after SPI in mice fed SD or MD-1. Right: representative images of incised paws from mice fed SD (left) and MD-1 (right). (K) CFA model: mice were fed SD or MD-1 for 21 days before hind-paw CFA injection. MD-1 access was extended for 1 week after injection, and behavior was tracked for 3 weeks. (L and M) Time-course of CFA-induced ipsilateral hypersensitivity (L, mechanical; and M, thermal) in mice fed SD (red triangles) or MD-1 (blue triangles). (B–E and G–M) Two-way ANOVA with Dunnett’s multiple comparisons test (*p < 0.05, **p < 0.01, and ***p < 0.001 vs. SD group; *n* = 8–10 per group).

MD1’s marked impact on formalin-induced pain encouraged us to explore the generalizability of its effects and their potential clinical value. To this end, we assessed MD-1’s efficacy in four chronic pain models: three involving damage to either nerve (CCI and SNI)^29,30^ or paw tissue (SPI),^31^ and one involving immune stimulation (CFA).^63^ We performed CCI or SNI in male SD- or MD-1-fed mice and monitored pain-related responses for the following 21 days, extending access to MD-1 for an additional week post-surgery (Figure 7A). In SD-fed mice, CCI and SNI caused lasting ipsilateral hypersensitivity, while MD-1-exposed mice were substantially protected (Figures 7B–7E). Moreover, SNI mice exhibited long-term memory deficits, which were also prevented by MD-1 (Figure S18A). Similarly, SD-fed mice subjected to SPI (Figure 7F) developed ipsilateral hypersensitivity, although of shorter duration (<2 weeks) than CCI or SNI (Figures 7G and 7H). This painful state was markedly attenuated by MD-1 exposure (Figures 7G and 7H). MD-1 also normalized the nocifensive response to prostaglandin E_2_ (PGE_2_) administered on post-surgical day 14 (Figure 7I), indicating that nociceptive priming^64^ was blocked. Interestingly, paw healing was accelerated in mice receiving MD-1 (Figure 7J), suggesting an overall strengthening of the animals’ resilience to tissue damage. In contrast, MD-1 did not influence CFA-induced pain (Figure 7K), producing only a small attenuation on day 7 after CFA administration (Figures 7L and 7M). To explore possible cellular substrates underpinning MD-1’s effects, we quantified by immunofluorescence glial fibrillary acidic protein (GFAP), ionized calcium-binding adapter molecule 1 (IBA-1), and SIRT1 in the lumbar spinal cord of mice fed either SD or MD-1 for 3 weeks. These analyses revealed that exposure to MD-1 normalized GFAP immunoreactivity, which was enhanced by formalin (Figure S18B), but produced only a small, not statistically significant effect on IBA-1 immunoreactivity (Figure S18C). Additionally, MD-1 increased the overall immunoreactive SIRT-1 levels in the lumbar spinal cord of both formalin- and vehicle-treated mice (Figures S19A and S19B) and normalized them in neurons and other cells following formalin injection (Figures S19B and S19C). Thus, MD-1 prevents persistent painful states induced by somatic injury, but not by immune triggers, through a mechanism that involves spinal cord neurons and astrocytes.

## DISCUSSION

The results offer several insights into the molecular mechanisms underlying the progression to pain chronicity. First, we show that peripheral injury activates AKT/mTORC1 in afferent segments of the spinal cord, stimulating local biomass production, suppressing autophagy-mediated biomass reclamation, and depleting vulnerable pools of amino acids and fatty acids. This metabolic crisis is exacerbated by concomitant activation of the PEA-degrading enzyme N-acylethanolamine acid amidase,^26,65^ which lowers PEA-mediated signaling at PPAR-α,^66,67^ aggravating mitochondrial dysfunction^68^ and promoting neuroinflammation.^69^ Second, experiments with the modified diet MD-1 reveal that this combined depletion of nutrients and PEA is a driving force behind chronic pain. Third, we show that the nutrient sensor SIRT1 controls pain processing in the spinal cord and—along with AMPK, which was previously implicated in chronic pain^70,71^—mediates the protective effects of MD-1. Fourth, we show that autophagy is a critical regulator of spinal pain processing and that deficits in this process enable minor injuries to cause enduring pain. Lastly, we demonstrate that the stimulation of apoptosis by injury, which was previously reported,^72^ is not directly involved in pain chronification. Collectively, these findings identify metabolic alterations in the spinal cord that drive pain chronification and suggest pharmacological and nutritional strategies to halt this process.

Previous work has shown that peripheral tissue damage induces central sensitization and persistent pain by recruiting AKT and its downstream target, mTORC1.^23–25^ Our results outline the molecular cascade linking AKT/mTORC1 activation to chronic pain development. They demonstrate that AKT/mTORC1 signaling promotes the transcriptional upregulation of glycolysis-related genes, increasing the levels of glycolytic metabolites, which provide fuel for protein and lipid biosynthesis in support of neuroplasticity.^17–21^ Concomitantly, SIRT1 and AMPK activities are downregulated, autophagy is suppressed, and critical amino acids, fatty acids, and PEA become depleted. Two key observations underscore the functional impact of this depletion: firstly, correcting the nutrient/PEA deficit with MD-1 stops the molecular cascade initiated by injury and interrupts pain chronification; secondly, inhibiting autophagy negates the protective effects of MD-1 and allows minor injuries to transition into lasting pain. These results emphasize the central role of core metabolism in chronic pain development, while raising several critical questions for future research: Which extracellular signals initiate injury-induced metabolic reprogramming? Which cell types release these signals, and which ones respond? How does metabolic reprogramming reshape neuroglial structure in the spinal cord to consolidate a painful state? Answering these questions will illuminate critical mechanistic aspects of the transition to pain chronicity and facilitate therapeutic discovery.

We designed MD-1 to augment the daily intake of specific amino acids, fatty acids, and PEA, which are depleted in the spinal cord following peripheral injury. Pharmacokinetic studies show that MD-1 is systemically bioavailable and prevents this depletion. Replenishing the reduced nutrients normalizes energy metabolism and biomass generation, reinstates autophagy, and stops chronic pain development. Mechanistic investigations reveal three significant features of these effects. First, the effects exhibit dose- and time-dependence, indicating that MD-1 interferes with specific steps of the molecular cascade triggered by injury. Second, the effects rely on a synergistic interaction between MD-1’s constituents: neither an isocaloric diet enriched solely with amino acids and fatty acids (MD-4) nor one supplemented exclusively with PEA (MD-5) provides more than partial and transient protection. Interestingly, at doses significantly exceeding those in MD-1, PEA, like other PPAR-α agonists, can circumvent the need for nutrient supplementation, directly inhibiting pain chronification and underscoring the pivotal regulatory function of PPAR-α in this process.^26^ Third, the effects of MD-1 involve multiple cell types in the spinal cord, including neurons and astrocytes, while microglia appear to play a minor role. Lastly, MD-1 enhances SIRT1 expression and AMPK activity—both of which are suppressed in the spinal cord of injured mice—while pharmacological activation of SIRT1 or AMPK replicates most, though not all, of MD-1’s effects. These findings highlight the integral role of SIRT1 and AMPK in modulating injury responses and mediating the therapeutic benefits of MD-1.

The best-understood function of SIRT1 and AMPK is to align core metabolism with energy availability.^32,33,62,73^ Notably, the most distinctive effects of these nutrient sensors are reminiscent of those produced by MD-1. For example, in liver and skeletal muscle, SIRT1 engages PPAR-α and PGC1α, among other targets, to suppress glycolysis and lipid biosynthesis and promote fatty acid oxidation and ketogenesis.^74–77^ Additionally, by interacting with mTORC1 and autophagosome component LC3B, SIRT1 represses protein translation and enhances autophagy.^78^ Similarly, AMPK downregulates anabolic processes and upregulates autophagy.^62^ AMPK also forms a positive feedback loop with SIRT1 in which AMPK recruits SIRT1 by upregulating the NAD^+^-synthetic enzyme nicotinamide phosphoribosyltransferase,^79^ while SIRT1 recruits AMPK by deacetylating its upstream kinase, liver kinase B1.^80^ Our studies show that activation of SIRT1 and AMPK effectively prevents pain chronification following injury. Interestingly, SIRT1 activation may also account for two effects of MD-1 that appear unrelated to the correction of injury-induced alterations in the spinal cord, namely, the diet’s ability to attenuate the acute nocifensive and inflammatory reaction to formalin and accelerate wound healing.^81,82^ These findings highlight a system-wide impact of MD-1 and suggest its potential applicability to a broad range of diseases, including age-related pathologies where SIRT1 and AMPK downregulation is implicated.^83–85^ Further studies are needed to explore these possibilities and clarify the precise mechanism through which SIRT1 and AMPK modulate pain chronification.

We conducted our mechanistic experiments in mice subjected to hind-paw formalin injection. We chose this model for three reasons. Firstly, while typically employed to study acute nociception,^36^ it also produces a lasting phenotype that replicates key features of severe chronic pain in humans—such as contralateral sensitization and structural reorganization of the forebrain^26^—features not fully replicated by other models.^29–31,63^ Secondly, the graded nature of the formalin response provides a valuable framework for investigating the factors that either facilitate or repress pain chronification. Here, we leveraged this property to evaluate the roles of autophagy suppression and SIRT1 or AMPK activation. Thirdly, prior studies have identified a critical window for pain chronification in this model,^26^ which we selected for our transcriptomic and metabolomic analyses. To evaluate the generalizability and translational relevance of MD-1’s effects, we tested the diet in four additional pain models: SNI, which involves partial sciatic nerve transection^29^; CCI, mimicking sciatic nerve compression and inflammation^30^; SPI, inducing inflammation and sensitization through paw incision^31^; and CFA, characterized by immune-driven pain without direct nerve injury.^63^ MD-1 demonstrated robust efficacy across all models except CFA, reinforcing the external validity of our findings and identifying post-surgical pain as a plausible clinical indication. The lack of effect in the CFA model confirms the existence of mechanistic differences between injury-induced and immune-driven pain,^86^ warranting further investigation.

In conclusion, our findings indicate that injury induces, via AKT/mTORC1 activation, metabolic reprogramming, biomass production, and autophagy suppression. These processes converge to cause a metabolic crisis that depletes key nutrients and PEA in the spinal cord and drives the transition to chronic pain, most likely by disrupting normal neuroplasticity. MD-1’s efficacy in halting this transition underscores the critical role played by core metabolism in this process. By replenishing amino acids, fatty acids, and PEA, MD-1 enhances SIRT1 and AMPK activity, re-establishes autophagy, and prevents pain chronification across multiple mouse models. Thus, the results not only reveal critical mechanistic factors driving the progression to pain chronicity but also provide a basis for the clinical application of nutritional interventions targeting this progression after invasive surgeries^3,4^ or accidental physical trauma.^5–7^

### Limitations of the study

This study has three main limitations. First, it was conducted in mice, and its results cannot be directly extrapolated to humans due to marked differences in the way these species respond to metabolic challenges.^87^ Nevertheless, the findings offer valuable insights to guide future clinical research: for example, MD-1’s efficacy in mouse models of post-surgical pain, combined with its lack of interference with wound healing, highlights its potential utility in perioperative analgesia. Moreover, the partial overlap between metabolomic changes in mouse serum and molecular events in the spinal cord suggests a possible strategy for identifying serum biomarkers of chronic pain progression in humans. Second, our experiments primarily focused on the spinal cord, leaving other potential target organs unexamined. Future investigations should explore the involvement of additional sites, including first-order nociceptors, immune cells, and metabolic organs, which may contribute to the systemic response to the bioenergy challenges posed by injury. Lastly, although the study uncovered important tissue-level changes in the spinal cord, it did not identify the specific cell types involved in these alterations. Elucidating these cellular mechanisms is a critical step toward the development of targeted interventions that interrupt or reverse pain chronification at its source.

## RESOURCE AVAILABILITY

### Lead contact

Further information and requests for resources and reagents should be directed to and will be fulfilled by the lead contact, Dr. Daniele Piomelli (piomelli@hs.uci.edu).

### Materials availability

This study did not generate new, unique reagents.

### Data and code availability

- RNA-seq and metabolomic data have been deposited in the NCBI Sequence Read Archive and MassIVE and are publicly available as of the date of publication. Accession numbers are listed in the key resources table. Data S1 refers to unprocessed data underlying the display items in the manuscript, related to all main and supplemental figures.
- This paper does not report original code.
- Any additional information required to reanalyze the data reported in this paper is available from the lead contact upon request.

## STAR★METHODS

### EXPERIMENTAL MODEL AND STUDY PARTICIPANT DETAILS

#### Animals

We used male and female CD-1 mice (7 weeks of age upon arrival; Charles River, Wilmington, MA). The mice were housed in single-sex groups of 4–5 per cage under a 12-hour light/dark cycle (lights on from 06:30 to 18:30) at controlled temperature (20 ± 2°C) and relative humidity (55–60%). They had *ad libitum* access to food and water. Upon arrival, the mice were acclimated to the animal facility for one week and subsequently fed either a control standard diet (SD) or a modified diet (MD) for varying amounts of time. The study followed ethical guidelines for laboratory animal care set by the National Institutes of Health (NIH) and the International Association for the Study of Pain (IASP). Experimental procedures were approved by the Animal Care and Use Committee of the University of California, Irvine (AUP-23–082).

### METHOD DETAILS

#### Chemicals

We purchased isoleucine, valine, tyrosine, methionine, and complete Freund’s adjuvant from Sigma Aldrich (Saint Louis, MO). A proprietary water-soluble PEA formulation (Levagen^+®^) was a kind gift of Gencor Pacific (Austin, TX). Metformin, [^2^H_4_]-PEA, alanine, proline, serine, threonine, cysteine, tryptophan, phenylalanine, and leucine were from Cayman Chemicals (Ann Arbor, MI). Oleic acid and erucic acid were from Nu-Chek Prep (Elysian, MN). Torin-1, MK-2206, SRT-2104, 3-methyladenine, emricasan, EX-527 and bafilomycin A1 were from MedChemExpress (Monmouth Junction, NJ). All other chemicals were obtained from Sigma Aldrich (Saint Louis, MO) or Honeywell (Muskegon, MI, USA) and were of the highest available grade.

#### Modified diets

MD-1 and other experimental diets were formulated by coarsely grinding a standard mouse diet (SD; Envigo 2020x) using a commercial mixer. The resulting fine powder was enriched with laboratory-grade compounds specifically selected to address the depletion of key substances in the ipsilateral L4-L6 spinal cord observed on days 3–4 following hind-paw formalin injection (Table S1). This enriched mixture was thoroughly blended with distilled water for 45 minutes to ensure uniformity. The prepared mixture was shaped into pellets using a pastry bag and extruded onto trays, followed by dehydration at room temperature for two days. The dried pellets were then stored in hermetically sealed containers, wrapped in tin foil to protect them from light and moisture. All diets were used within 30 days of preparation to ensure freshness and stability.

#### Drug administration

Drug solutions were freshly prepared prior to each use and administered via intraperitoneal (IP) or subcutaneous (SC) injection, depending on the experimental protocol. Metformin was dissolved in sterile saline. Torin-1, MK-2206, SRT-2104, emricasan, EX-527, TMA, and bafilomycin A1 were dissolved in a vehicle consisting of polyethylene glycol 400/Tween 80/saline (15:15:70, vol).

#### Pain models

##### Formalin

We injected a diluted formalin solution (1% or 0.1% in sterile saline, 20 μL) or saline into the plantar surface of the right hind paw, as described.^88^ Following injections, the mice were immediately transferred to a transparent observation chamber where nocifensive behaviors (time spent licking or biting the injected paw and number of paw shakings) were videorecorded for 60 min to be later quantified by an observer blinded to experimental conditions. Mechanical hypersensitivity, heat hypersensitivity, and paw edema were measured at various time points in both formalin-injected and noninjected hind paws, as detailed under *Behavioral tests*.

##### Spared nerve injury

We used a protocol described previously.^89,90^ Briefly, the mice were anesthetized with 2–3% isoflurane in O_2_ delivered via a face mask. The right common sciatic nerve was exposed under aseptic conditions by blunt dissection at the level of its trifurcation into sural, tibial, and common peroneal nerves. The common peroneal and tibial branches of the sciatic nerve were tightly ligated with a 5.0 silk suture and transected distally, while the sural nerve was left intact. The wound was closed with a single muscle suture and skin clips. In sham-operated animals, the sciatic nerve was exposed but not transected.

##### CCI

Chronic constriction injury (CCI) of the sciatic nerve was carried out as described.^88^ Briefly, the mice were anesthetized with 2–3% isoflurane in O_2_ delivered via a face mask. The right common sciatic nerve was exposed under aseptic conditions at the level of the middle thigh by blunt dissection. Proximal to the trifurcation, the nerve was cleaned from surrounding connective tissue, and three chromic catgut ligatures (4–0, Ethicon, Somerville, USA) were loosely tied around it at 1-mm intervals. The wound was closed with a single muscle suture and skin clips. In sham-operated animals, the sciatic nerve was exposed but not tied.

##### SPI

We performed surgical paw incision (SPI) as described.^91,92^ Briefly, the mice were anesthetized with 2–3% isoflurane in O_2_ delivered via a face mask. A 0.5-cm longitudinal incision was made under aseptic conditions through the skin and fascia of the plantar aspect of the right hind paw using a scalpel blade. The incision started 0.2 cm from the proximal edge of the heel and extended distally. The plantaris muscle was elevated with curved forceps and incised longitudinally, leaving the muscle origin and insertion intact. After hemostasis, the skin was sutured with a 6–0 nylon on a FS-2 needle (Ethicon, USA) and an antibiotic ointment was applied. Unwounded mice underwent a sham procedure consisting of anesthesia, antiseptic preparation, and ointment application. Following surgery, mice were returned to their home cages and continued receiving either SD or MD-1. Wound healing was monitored as described,^92^ assigning a 1-point score to each of the following parameters: heat hypersensitivity, mechanical hypersensitivity, redness, visible edema, presence of pus, wound closure, and scar formation.

##### HP

Hyperalgesic priming (HP) was induced using the paw surgical incision protocol described above. Following surgery, mice were returned to their home cages. On day 14 post-surgical paw incision, prostaglandin E_2_ (PGE_2_; 100 ng in 20 μL) was administered subcutaneously into the lesioned paw. Nocifensive behavior (thermal hypersensitivity) was monitored for 6 hours post-injection.

##### CFA-induced inflammation

We injected complete Freund’s adjuvant (CFA) (1 mg-mL^−1^, 20 μL) into the plantar surface of the right hind paw of slightly restrained mice.^93^ Nocifensive behavior (thermal and mechanical hypersensitivity) was assessed before the injection and on days 7, 14 and 21 post-injection.

#### Behavioral tests

##### Mechanical hypersensitivity

Mechanical hypersensitivity was evaluated using a dynamic plantar aesthesiometer (Ugo Basile, Italy).^94^ After a 45-min habituation period in transparent cages positioned on a wire mesh surface, a mechanical stimulus was applied to the plantar surface of both hind paws by an automated steel filament exerting a force increasing from 0 to 5 g over 10 s. Withdrawal threshold was defined as the force (in grams) at which mice withdrew their paws from the mechanical stimulus. Three measurements were taken at intervals of 3 min and averaged.

##### Heat hypersensitivity

Sensitivity to heat was measured using a Hargreaves plantar test apparatus (San Diego Instruments, San Diego, USA) as described.^94,95^ After a 45-min habituation period, the plantar surface of both hind paws was exposed to a beam of radiant heat through the glass floor. The cutoff time was set at 15 s. The stimulation was repeated three times with an interval of 2 min between stimuli, and latencies (in seconds) to withdraw the paw were recorded and averaged.

##### Tail flick

Tail-flick assays were conducted following an established protocol.^94^ Mice were gently restrained in a soft tissue pocket made of pet-training pad (Glad™), and the distal 1/3 of each mouse’s tail was immersed in a hot water bath maintained at 54°C. The latency to withdraw the tail from the bath was recorded (in seconds). Measurements were performed twice, separated by a 5-min interval between trials, and the results were averaged. A 10-s cut-off time was implemented to prevent tissue damage.

##### Paw edema

Paw edema was measured with a digital caliper (Fisher Scientific, USA) and is expressed as the difference (Δ thickness, mm) between ipsilateral and contralateral paws.

##### Elevated plus maze

The test was performed under low ambient lighting (open arms: 160–180 lux and closed arms: 40–50 lux in accordance with an established protocol.^96^ Briefly, each mouse was placed on the central platform of the maze, facing closed open arms, and the trial was recorded for 5 min using the Debut video capture software (NCH Software, Canberra, Australia). A blinded observer measured the time spent in the open and closed arms, as well as the number of entries into each arm type. The anxiety index was calculated using the formula: 1 - [(time spent in open arms/total time) + (open arm entries/total entries)] / 2.^26^

##### Novel object recognition

The test was conducted over 3 days.^26,97^ On day 1, the mice were acclimated to the empty arena for 10 min. On day 2, they were reintroduced to the arena, which now contained two identical objects, and were left there for 10 min. On day 3, one of the objects was substituted with a new object of different shape, color, and texture. Mice were given another 10-min session to explore the arena, during which an observer blinded to experimental conditions recorded the total time spent exploring each object (i.e., nosing and sniffing at a distance ≤ 2 cm). The discrimination index was computed as: [(time of novel object exploration) – (time of familiar object exploration)]/total exploration time.

#### Tissue collection

Mice were deeply anesthetized with isoflurane. Blood was collected via cardiac puncture using syringes either rinsed with ethylene-diaminetetraacetic acid (EDTA) or left unrinsed, and transferred into 1 mL polypropylene tubes containing either spray-coated potassium-EDTA (for plasma collection) or no anticoagulant (for serum collection). The blood was centrifuged at 1,450 × *g* for 15 min at 4°C. The resulting supernatant (serum or plasma) was carefully transferred into polypropylene tubes, immediately frozen, and stored at −80°C. Spinal cords were harvested by gentle hydraulic extrusion onto an ice-cold glass plate. The ipsilateral L4-L6 lumbar segments were dissected, snap-frozen on dry ice, and stored at −80°C until further analysis.

#### RNA sequencing and bioinformatics analysis

We extracted total RNA from L4-L6 spinal cord segments with the RNeasy Mini Kit (Qiagen) following manufacturer’s instructions. Samples with RNA integrity number (RIN) ≥ 8.5 were used for library construction. cDNA synthesis, amplification, library construction, and sequencing were performed at Novogene (Beijing, China) using the Illumina NovaSeq platform with paired-end 150–base pair sequencing strategy. Downstream bioinformatic analyses were performed using a combination of programs including STAR, HTseq, Cufflink and Novogene’s wrapped scripts, and alignments were parsed using STAR. Principal component analysis (PCA) and comparative analyses of differentially expressed genes (DEGs) were performed using the DESeq2/edgeR package and a model based on negative binomial distribution. Resulting *P* values were adjusted using the Benjamini and Hochberg’s approach for controlling false discovery rate (adjusted *P* values, *Padj*). Comparative analysis of DEGs was carried out between two test groups. Changes displaying *Padj* < 0.05 were considered significant. DEG distribution was assessed using Volcano plots showing statistical significance (*Padj*) vs magnitude of change (fold change). DEGs were annotated using the Database for Annotation, Visualization and Integrated Discovery (DAVID) database, PANTHER gene ontology (GO) knowledge base, and the Kyoto Encyclopedia of Genes and Genomes (KEGG) pathway database, which was implemented using the ShinyGO 0.80 bioinformatics platform. GO terms with *Padj* < 0.05 were considered significantly enriched in DEGs.

#### Metabolomic analyses

L4-L6 spinal cord segments were harvested and snap frozen on dry ice. Samples were pulverized to a homogeneous powder using a Cryomill (Retsch, Newtown, PA). An ice-cold mixture of methanol:acetonitrile:water (40:40:20, vol; 0.5–0.6 mL) was added to ∼10 mg of the powdered samples to make 25 mg/mL suspensions, which were centrifuged at 16,000 × *g* for 10 min at 4°C. For serum, 5 μL were diluted 30-fold with the same ice-cold mixture of methanol:acetonitrile:water and centrifuged under the same conditions. Supernatants (3 μL) from spinal cord and serum samples were analyzed as described.^98^ Briefly, a quadrupole-orbitrap mass spectrometer (Q Exactive Plus, ThermoFisher Scientific) operated in negative or positive ionization mode was coupled to a Vanquish Ultra High-Performance LC system (Thermo Fisher Scientific) with electrospray ionization. Scan range was *m/z* 70–1000, scanning frequency was 2 Hz and resolution was 140,000. LC separations were conducted using a XBridge BEH Amide column (2.1 mm × 150 mm^2^, 2.5 μm particle size, 130Å pore size pore size; Waters Corporation) with a gradient consisting of solvent A (20 mM ammonium acetate, 20 mM ammonium hydroxide in 95:5 water:acetonitrile, pH 9.45) and solvent B (acetonitrile). The flow rate was 0.150 mL/min. The gradient was: 0 min,85% B; 2 min, 85% B; 3 min, 80% B; 5 min, 80% B; 6 min, 75% B; 7 min, 75% B; 8 min, 70% B; 9 min, 70% B; 10 min, 50% B; 12 min, 50% B; 13 min, 25% B; 16 min, 25% B; 18 min, 0% B; 23 min, 0% B; 24 min, 85% B; 30 min, 85% B. Autosampler temperature was 5°C. Data were analyzed using the MAVEN software (Build # 682), Compound Discoverer software (Thermofisher Scientific), and R software. To control for instrument variability, an internal control [^13^C_5_, ^15^N]-valine, was spiked in the extraction solvent.

#### PEA analysis

##### PEA extraction

We extracted PEA from plasma and spinal cord samples as described.^97^ Briefly, plasma (0.1 mL) was transferred into 8-mL glass vials. Proteins were precipitated by the addition of 0.45 mL ice-cold acetonitrile containing 1% formic acid and 0.05 mL internal standard [^2^H_4_]-PEA. The mixture was stirred vigorously for 30 s and centrifuged at 1450 × *g* at 4°C for 15 min. Spinal tissue samples (∼20 mg each) were transferred into 2 mL Precellys CK-14 soft tissue tubes (Bertin, Rockville, MD) and homogenized in 0.5 mL of ice-cold acetonitrile containing 1% formic acid and the internal standard listed above. Supernatants from plasma or spinal tissue samples were loaded onto Enhanced Matrix Removal (EMR)-Lipid cartridges (Agilent Technologies, Santa Clara, CA) and eluted under positive pressure (3–5 mmHg, 1 drop/5 sec). Residual pellets from plasma and spinal tissue were rinsed with water/acetonitrile (1:4 vol/vol; 0.2 mL), stirred for 30 s, and centrifuged at 1450 × *g* at 4°C for 15 min. The supernatants were transferred onto EMR cartridges, eluted (1 drop/sec), and pooled with the first eluate. The cartridges were rinsed with water/acetonitrile (1:4 vol/vol; 0.2 mL), and pressure was gradually increased to 10 mmHg for maximal analyte recovery. Eluates were dried under a gentle stream of N_2_ (2 mmHg for 1 hour), reconstituted in methanol (0.1 mL) and transferred to deactivated glass inserts (0.2 mL) placed inside amber glass vials (2 mL, Agilent Technologies) for liquid chromatography/tandem mass spectrometry (LC-MS/MS) analyses.

##### PEA quantification

We quantified PEA using a 1260 series LC system (Agilent Technologies, Santa Clara, CA) coupled to a 6460C triple-quadrupole mass spectrometry detector (MSD; Agilent). An Eclipse PAH column (1.8 μm, 2.1 × 50 mm; Agilent Technologies) was eluted with a mobile phase consisting of 0.1% formic acid in water as solvent A and 0.1% formic acid in methanol as solvent B. A linear gradient was used: 75.0% B at 0 time to 80.0% B in 5.0 min, change to 95% B at 5.01 min continuing to 6.0 min, change to 75% B at 6.01 min, and hold till 8.0 min for column re-equilibration and stop time. The column temperature was maintained at 45°C and the autosampler temperature at 10°C. The injection volume was 2 μL, the flow rate was 0.3 mL/min, and the total analysis time was 15.5 min. To prevent carryover, the injection needle was washed three times in the autosampler port for 30 s before each injection, using a wash solution consisting of 10% acetone in water/methanol/isopropanol/acetonitrile (1:1:1:1, vol). The MSD was operated in the positive electrospray ionization (ESI) mode. PEA was quantified by multiple reaction monitoring (MRM) of the following transitions: PEA = *m/z* 300.3 > 62.2, [^2^H_4_]-PEA = *m/z* 304.3 > 66.2. The lowest limit of detection (LOD) was 0.5 ng/mL; lowest limit of quantification (LOQ) was 1 ng/mL. Capillary and nozzle voltages were 3,000 and 1,900 V, respectively. The drying gas temperature was 300°C with a flow of 5 mL/min. The sheath gas temperature was 300°C with a flow of 12 mL/h. Nebulizer pressure was set at 40 psi. We used MassHunter software version B.08.00 (Agilent Technologies) for instrument control, data acquisition, and analysis.

#### Protein analyses

##### Western blot

Western blot analyses were performed as described.^99^ L4–L6 lumbar spinal cord segments were dissected, snap-frozen in liquid nitrogen, and stored at −80°C. For protein extraction, tissues were homogenized on ice in RIPA lysis buffer (Thermo Fisher Scientific, Waltham, MA) containing a protease and phosphatase inhibitor cocktail (Thermo Fisher Scientific). Homogenates were centrifuged at 13,000 × g for 10 min at 4°C, and the supernatants were collected as total protein extracts. Protein concentrations were determined using the Pierce BCA Protein Assay Kit (Thermo Fisher Scientific). Equal amounts of protein (20–40 μg) were mixed with 4× Laemmli sample buffer (Bio-Rad, Hercules, CA) containing β-mercaptoethanol, heated at 95°C for 5 min, and separated on 4%−12% SDS-PAGE gels. Proteins were transferred to nitrocellulose membranes at 100 V for 90 min in transfer buffer containing 20% methanol. Membranes were blocked in 5% skim milk in Tris-buffered saline with 0.1% Tween-20 (TBST, pH 7.4) for 1 hour at room temperature. Membranes were then incubated overnight at 4°C with primary antibodies listed above. Following incubation with primary antibodies, membranes were washed three times with TBST and incubated for 1 hour at room temperature with species-specific HRP-conjugated secondary antibodies (1:5000, Cell Signaling Technology). Membranes were washed again with TBST and developed using an ECL detection kit (Thermo Fisher Scientific). Blots were visualized using Image Lab 6.1 software, and densitometric analysis of band intensities was performed using ImageJ software (National Institutes of Health). Protein expression levels were normalized to β-actin and expressed as fold changes relative to control.

##### Immunofluorescence

Mice were deeply anesthetized and transcardially perfused with ice-cold phosphate-buffered saline (PBS), followed by 4% paraformaldehyde (PFA) in PBS. Lumbar spinal cords (L4–L6 segments) were dissected, post-fixed in 4% PFA for 4–6 hours at 4°C, and cryoprotected overnight in 30% sucrose in PBS (pH 7.4) at 4°C. Tissues were embedded in optimal cutting temperature (OCT) compound (Tissue-Tek^®^, Sakura Finetek, Torrance, CA) and flash-frozen in cold isopentane. Cryosections (10 μm thickness) were prepared using a cryostat and mounted on Superfrost Plus slides (Cat. #1255015, Thermo Fisher Scientific). Sections were rinsed with 0.1 M phosphate buffer (PB) (76.43 mM Na_2_HPO_4_, 23.73 mM NaH_2_PO_4_, distilled water, pH 7.4), permeabilized with 0.3% Triton X-100 in PB, and blocked with 3% normal horse serum (NHS) in PB for 1 hour at room temperature. Immunostaining was performed by incubating sections overnight at 4°C with primary antibodies diluted in staining buffer containing 3% NHS and 0.3% Triton X-100 in 0.1 M PB as follows: rabbit anti-LC3B (1:1000), rabbit anti-Caspase-3 (1:1000), rabbit anti-SIRT1 (1:1000), mouse anti-GFAP (1:400), mouse anti-IBA1 (1:1000), mouse anti-NeuN (1:400). After three 10-minute washes in 0.1 M PB, sections were incubated at room temperature for 1 hour in the dark with Alexa Fluor 568-conjugated goat anti-rabbit secondary antibody (1:1000) diluted in PB containing 3% NHS and 0.3% Triton X-100. Sections were mounted using VECTASHIELD^®^ Antifade Mounting Medium with DAPI (Cat. #H1200, Vector Laboratories, Newark, CA) and sealed with coverslips. Images were captured at 10x, 20x, or 40x magnification using a Keyence BZ-X810 fluorescence microscope. LC3B-, SIRT1-, GFAP-, IBA1- or Caspase-3-positive puncta were analyzed using ImageJ software, and neuronal localization was confirmed by co-staining with mouse anti-NeuN antibody (1:400). Quantification of LC3B- and Caspase-3-positive puncta was performed in at least three randomly selected sections.

#### Gut microbiome analysis

##### Fecal sample preparation

Mixed fecal droppings (∼2ml in volume) were collected from 4 mice in each cage (*n* = 4 cages per group). The samples were placed into 15-mL tubes and immediately stored at −80°C. They were thawed once for DNA extraction and 16S rRNA sequencing.

##### Preparation of DNA and 16S library construction

Extraction of DNA from frozen stool samples was performed using Zymo Research Quick-DNA Fecal/Soil Microbe 96 Magbead Kit according to manufacturer’s instructions. Approximately 180–200 mg of stool sample was used for the DNA extraction. The resulting DNA was measured by Qubit and 5 ng was used as input for library construction. The library preparation was performed according to the Illumina 16S Metagenomic Sequencing Library Preparation protocol. More specifically, the protocol is a two-step PCR that begins with the primer pair sequences for the V3 and V4 region with partial Illumina adapter handles to generate a single amplicon of approximately ∼460 bp [16S Amplicon Forward (V3 region): 5^′^-TCGTCGGCAGCGTCAGATGTGTATAAGAGACAGCCTACGGGNGGCWGCAG-3, and 16S Amplicon Reverse (V4 region): 5^′^-GTCTCGTGGGCTCGGAGATGTGTATAAGAGACAGGACTACHVGGGTATCTAA TCC-3’]. The second step PCR completes the Illumina adapter and adds P5 and P7 indexes using primers for from the dual index kit for Nextera XT library construction. The resulting libraries were assayed for quantity using Qubit and for quality using the Agilent Bioanalyzer 2100 DNA HS chip. The libraries were normalized and then multiplexed together. The multiplexed library pool was quantified using qPCR and sequenced on Illumina Miseq 2X300bp run.

##### Analysis

We imported 978,449 demultiplexed Illumina Miseq sequence reads into QIIME2 version 2022.2^100^ (https://qiime2.org). Quality checking, denoising, and merging of paired-end reads were performed using DADA2 via the q2-dada2 plugin.^101^ We picked operational taxonomic units (OTUs) at a 100% identity level (amplicon sequence variants) using UCLUST via QIIME2. We assigned taxonomy to the OTUs using the q2-feature-classifier, classify-sklearn naive Bayes taxonomy classifier against the SILVA database 138 against the OTUs reference sequences.^102,103^ The QIIME2-created OTU table as well as the taxonomy table and metadata were transferred into R for statistical analysis (R version 4.2.2). We rarefied the OTU table via randomized sampling without replacement with 300 iterations at 22,000 sequences per sample using the “EcolUtils” package (R core Team, 2018, https://www.r-project.org/; Salazar, G. 2020. EcolUtils: Utilities for community ecology analysis. https://github.com/GuillemSalazar/EcolUtils). We determined the effect of MD-1 on microbial composition was determined using Permutational multivariate analysis of variance (PERMANOVA) on a Bray Curtis dissimilarity matrix generated from the rarefied OTU table using the adonis2 function of the vegan package (version 2.6–4) in R. We performed a Shapiro-Wilk test to check for normality distribution of residuals for the Shannon diversity. Since the distribution was normal, an ANOVA was used to check for significance of any of the factors for alpha diversity.

### QUANTIFICATION AND STATISTICAL ANALYSIS

Statistical analyses were conducted using GraphPad Prism version 10.2 (La Jolla, CA). Results are presented as means ± SEM of n experiments. Statistical significance was set at *P* < 0.05 and assessed using unpaired, two-tailed Student’s *t* test or analysis of variance (ANOVA) (one-way or two-way) followed by post hoc tests, as appropriate. Analysis of transcriptomics and microbiome data were conducted as described in previous sections.

## Supplementary Material

1

2

3

4

5

Supplemental information can be found online at https://doi.org/10.1016/j.celrep.2025.116261.

## Figures and Tables

**Table:** KEY RESOURCES TABLE

REAGENT or RESOURCE	SOURCE	IDENTIFIER

Antibodies		

Rabbit polyclonal anti-p-AKT (Ser473)	Cell Signaling Technology	9271; RRID:AB_329825
Rabbit polyclonal anti-AKT	Cell Signaling Technology	9272; RRID:AB_329827
Rabbit monoclonal anti-p-mTOR (Ser2448)	Cell Signaling Technology	5536; RRID:AB_10691552
Rabbit polyclonal anti-mTOR	Cell Signaling Technology	2972; RRID:AB_330978
Rabbit monoclonal anti-p-AMPKa (Thr172)	Cell Signaling Technology	2535; RRID:AB_331250
Rabbit polyclonal anti-AMPKa	Cell Signaling Technology	5831; RRID:AB_10622186
Rabbit polyclonal anti-LC3B	Cell Signaling Technology	2775; RRID:AB_915950
Rabbit polyclonal anti-Caspase-3 (Asp175)	Cell Signaling Technology	9661; RRID:AB_2341188
Mouse anti-Iba1/AIF1	Millipore Sigma	MABN92; RRID:AB_10917271
Rabbit monoclonal anti-SIRT1 (D1D7)	Cell Signaling Technology	9475; RRID:AB_2617130
Mouse monoclonal anti-GFAP (GA5)	Cell Signaling Technology	3670; RRID:AB_561049
Mouse anti-NeuN (E4M5P)	Cell Signaling Technology	94403; RRID:AB_2904530
Alexa Fluor 568-conjugated donkey anti-rabbit	Thermo Fisher Scientific	A78946; RRID:AB_2910653
Alexa Fluor 488-conjugated goat anti-mouse	Thermo Fisher Scientific	A11029; RRID:AB_2534088
Mouse monoclonal anti-SIRT1	Abcam	ab110304; RRID:AB_10864359

Chemicals, peptides, and recombinant proteins		

Isoflurane	Pivetal veterinary	21295098
EDTA (0.5M), pH 8.0	Thermo Fisher Scientific	AM9260G
Acetonitrile	Thermo Fisher Scientific	T001014000
Methanol	Thermo Fisher Scientific	T001024000
Water, HPLC grade	Thermo Fisher Scientific	WSSK-4
Formic acid	Thermo Fisher Scientific	A117–50
Invitrogen TRIzol	Thermo Fisher Scientific	15596026
Isoleucine	Sigma Aldrich	I2752
Valine	Sigma Aldrich	V0500
Tyrosine	Sigma Aldrich	T3754
Methionine	Sigma Aldrich	M9625
Complete Freund’s Adjuvant (CFA)	Sigma Aldrich	F5881
PEA formulation (Levagen^®^+)	Gencor Pacific	PML0010815
Metformin	Cayman Chemicals	13118
Palmitoylethanolamide-d4 ([^2^H_4_]-PEA)	Cayman Chemicals	10007824
Alanine	Cayman Chemicals	29757
Proline	Cayman Chemicals	30772
Serine	Cayman Chemicals	33561
Threonine	Cayman Chemicals	30194
Cysteine	Cayman Chemicals	33309
Tryptophan	Cayman Chemicals	29600
Phenylalanine	Cayman Chemicals	31498
Leucine	Cayman Chemicals	34342
Oleic acid	Nu-Chek Prep	U-46-A
Erucic acid	Nu-Chek Prep	U-79-A
Torin-1	MedChemExpress	HY-13003
MK-2206	MedChemExpress	HY-10358
SRT-2104	MedChemExpress	HY-15262
3-methyladenine (TMA)	MedChemExpress	HY-19312
Bafilomycin A1 (bafA1)	MedChemExpress	HY-100558
Emricasan	MedChemExpress	HY-10396
EX-527	MedChemExpress	HY-15452
Formic acid	Thermo Fisher Scientific	28905
Antifade Mounting Medium with DAPI	Vector Laboratories	H1200
Protease inhibitor	Sigma-Aldrich	P8340

Critical commercial assays		

Quick-DNA Fecal/Soil Microbe 96 Magbead Kit	Zymo Research	D6012-FM
Pierce BCA Protein Assay Kit	Thermo Fisher Scientific	23227
RNeasy Mini Kit	Qiagen	74104

Deposited data		

RNA-seq	NCBI Sequence Read Archive	PRJNA1306173
Metabolomic dataset	MassIVE	MSV000098448

Experimental models: Organisms/strains		


Mouse: *CD-1*		


Software and algorithms		

ImageJ	https://imagej.nih.gov/ij/download.html	N/A
GraphPad Prism	GraphPad Prism	Version 10.2.3
MassHunter	Agilent Technologies	N/A
STRING	https://string-db.org/	N/A

## Highlights

- Bodily injury directs spinal metabolism to produce biomass in support of plasticity
- Critical nutrients in afferent spinal cord segments are depleted
- Dietary prevention of this depletion corrects metabolism and stops pain chronification
- Penury-responsive sensors, SIRT1 and AMPK, contribute to the diet’s protective effects
