# Supplementary material for: Metabolic reprogramming in the spinal cord drives the transition to pain chronicity
Source: Cell Rep. Author manuscript; Available in PMC 2025 Dec 20. (PMC12717780; doi:10.1016/j.celrep.2025.116261)
Supplement: 1 [file NIHMS2113405-supplement-1.pdf]

Cell Reports, Volume *44*

Supplemental information

Metabolic reprogramming in the spinal cord drives the transition to pain chronicity

Alex Mabou Tagne, Yannick Fotio, Hye-Lim Lee, Kwang-Mook Jung, Jean Katz, Faizy Ahmed, Johnny Le, Richard Bazinet, Cholsoon Jang, and Daniele Piomelli

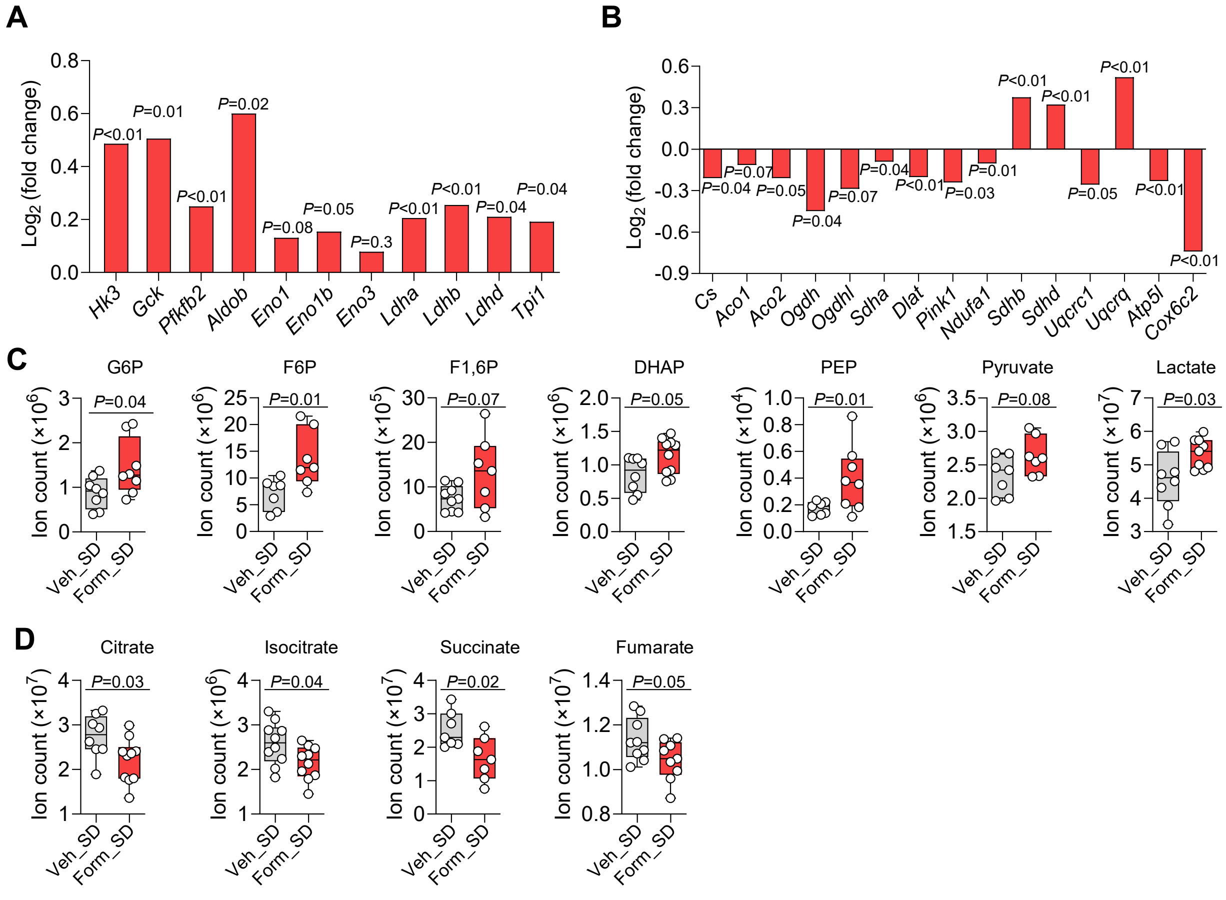

Figure S1. (Related to Figure 1) Effects of formalin injection on gene transcription and metabolite levels in ipsilateral L4-L6 spinal cord of SD-fed mice. (A, B) Transcription of genes involved in (A) glycolysis and (B) Krebs's cycle and oxidative phosphorylation. Data are expressed as log2 changes (formalin vs vehicle) (*n* = 6; multiple unpaired t test). (C, D) Concentrations of (C) glycolysis and (D) Krebs's cycle metabolites. Data are expressed as ion counts (mean ± SEM; *n* = 7–10 per group; Student's t test).

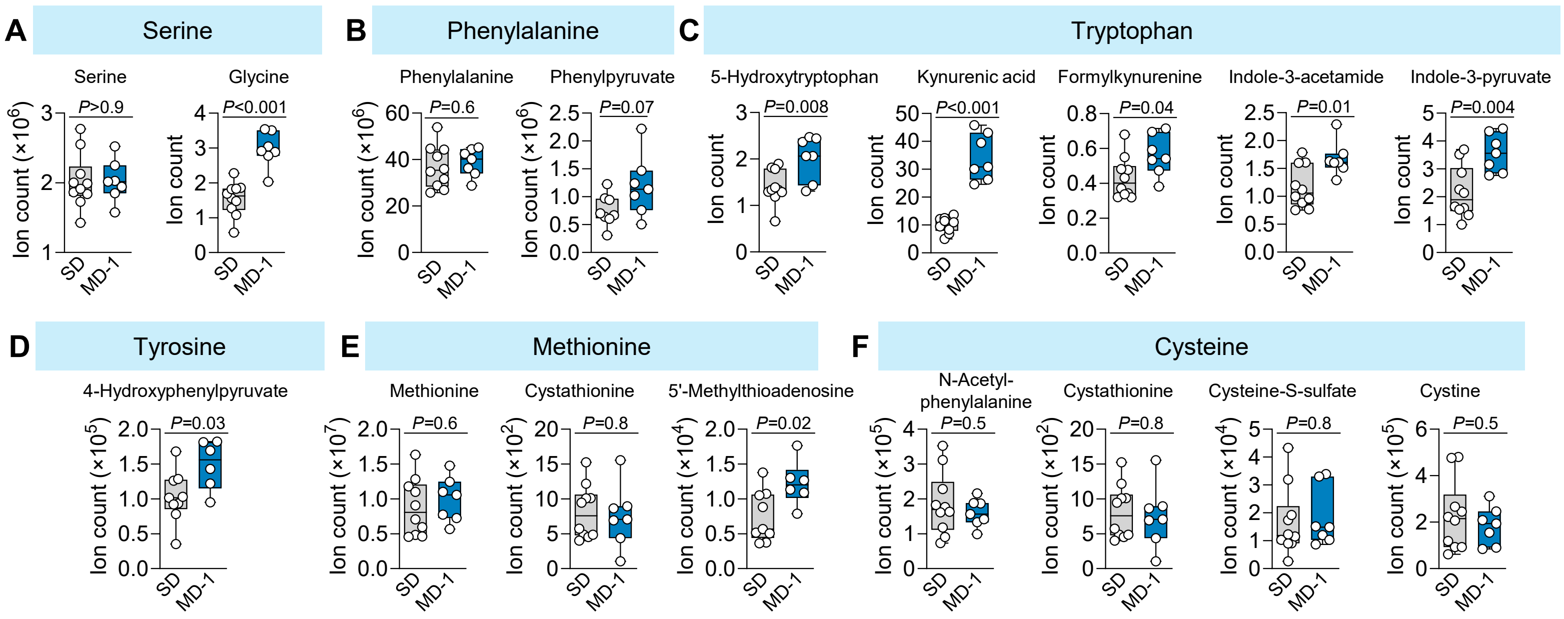

Figure S2. (Related to Figure 1B) Serum concentrations of amino acids and their metabolites in vehicle-injected mice fed SD (gray boxes; *n* = 9-10) or MD-1 (blue boxes; *n* = 6-7) for 25 days. A) Serine and metabolites; B) Phenylalanine and metabolites; C) Tryptophan and metabolites; D) Tyrosine; E) Methionine and metabolites; F) Cysteine and metabolites. Data are expressed as ion counts (mean ± SEM; Student's *t* test).

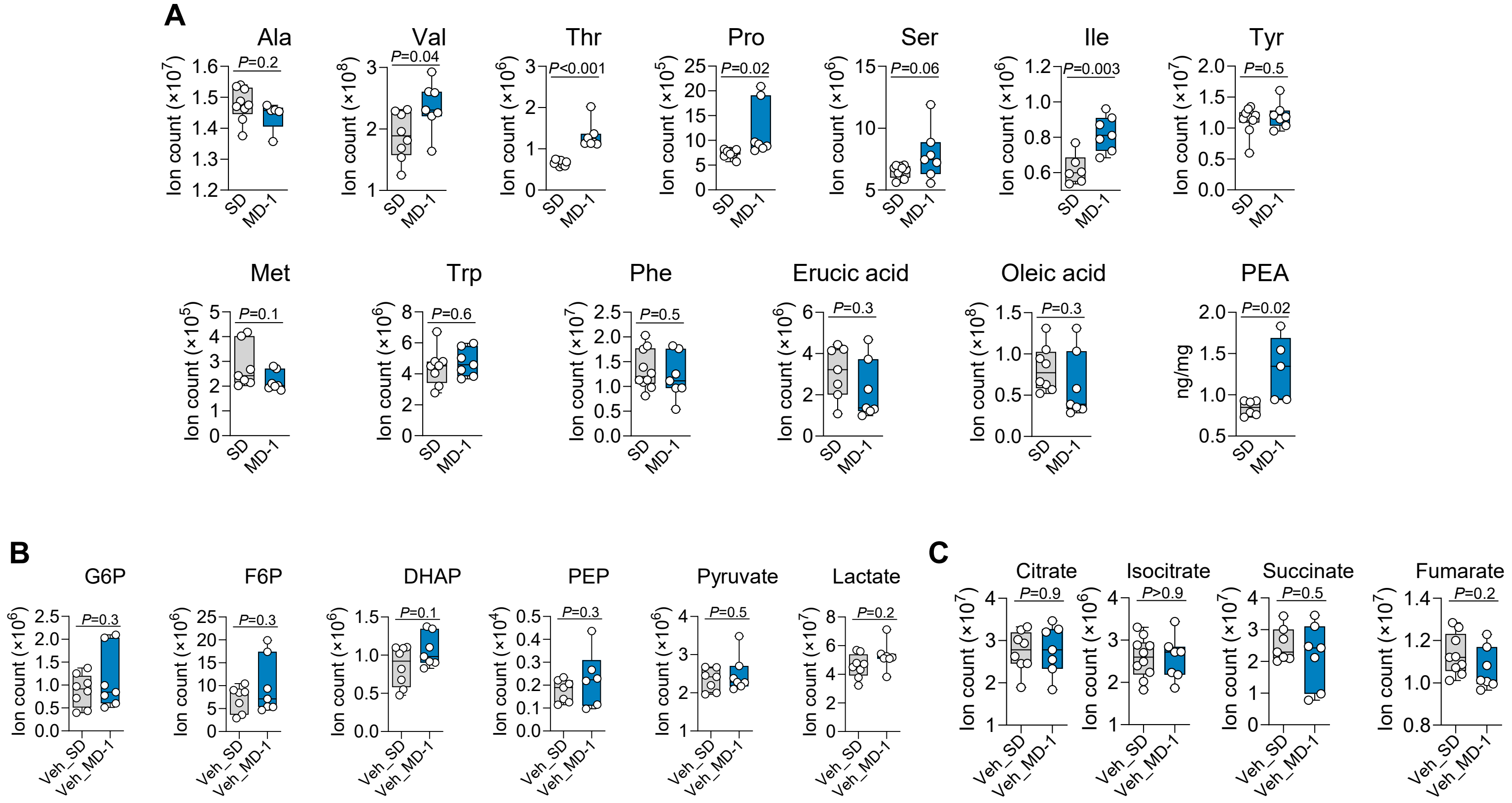

Figure S3. Concentrations of MD-1 components and metabolites in the ipsilateral L4–L6 spinal cord of vehicle-injected mice fed SD or MD-1 for 25 days. (A) (Related to Figure 1D, E) Concentrations of MD-1 components in ipsilateral L4-L6 spinal cord of vehicle-injected mice fed SD (gray boxes; *n* = 7-10) or MD-1 (blue boxes; *n* = 5-7) for 25 days. Data are expressed as ion counts (mean ± SEM; Student's t test). (B, C) (Related to Figure 2) Concentrations of (B) glycolysis and (C) Krebs' cycle metabolites in ipsilateral L4-L6 spinal cord of vehicle-injected mice fed SD (gray boxes) or MD-1 (blue boxes). Data are expressed as ion counts (mean ± SEM; *n* = 7-10 per group; Student's t test).

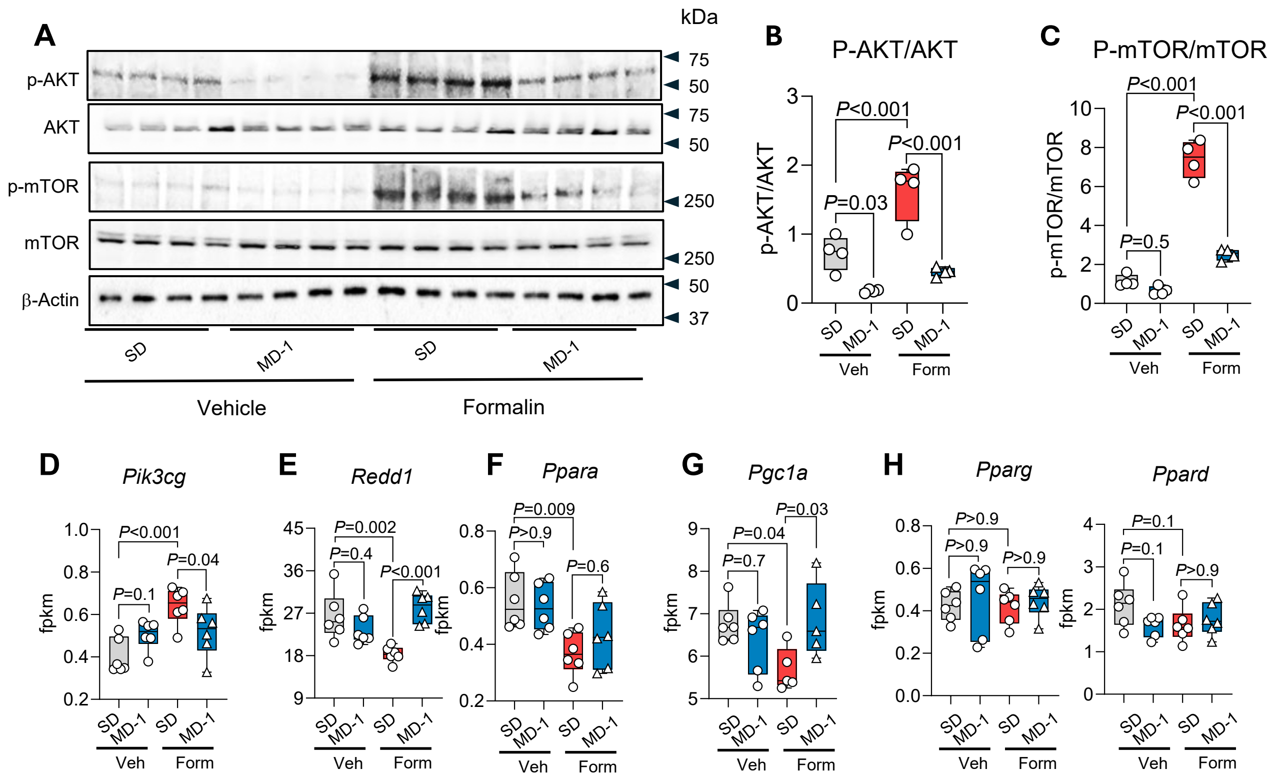

Figure S4. (Related to Figure 3) Formalin injection activates AKT/mTORC1 in ipsilateral L4-L6 spinal cord and MD-1 counters this activation. (A) Representative Western blot images showing levels of phospho-AKT (p-AKT), AKT, phospho-mTOR (p-mTOR), and mTOR in vehicle- or formalin-injected mice fed SD or MD-1. β-actin is the loading control. (B, C) Quantification of phospho-AKT (p-AKT/AKT) and phospho-mTOR (p-mTOR/mTOR) in vehicle (Veh)- or formalin (Form)-injected mice fed SD or MD-1. (D-H) Transcription of (D) *Pik3cg*, (E) *Redd1*, (F) *Ppara*, (G) *Ppargc1a* (*Pgc1a*), and (H) *Pparg* and *Ppard*. Data are expressed as mean ± SEM (n = 4-6 per group); one-way ANOVA with post hoc Šídák's test. *P<0.05, **P<0.01, ***P<0.001 compared to formalin/SD.

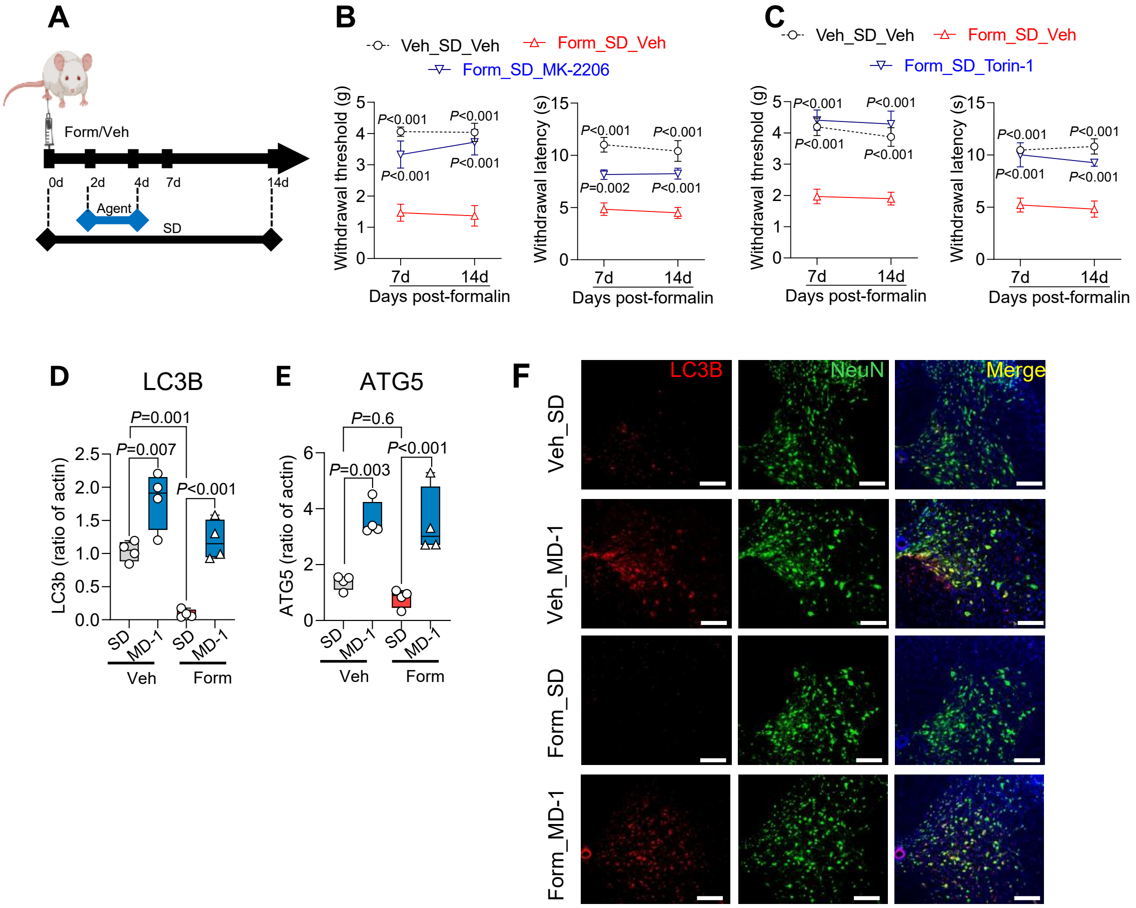

Figure S5. Post-injury inhibition of AKT and mTOR pathways reduces contralateral hypersensitivity and modulates spinal autophagy markers in formalin-injected mice. (A-C) (Related to Figure 3) Effects of post-injury administration of AKT and mTOR inhibitors in formalin-injected mice. (A) Protocol: SD-fed mice were treated with AKT inhibitor MK-2206 (240 mg/kg, IP) or mTOR inhibitor Torin-1 (20 mg/kg, IP) on days 2-4 post-injection. Nocifensive behavior was monitored for the following two weeks. (B, C) Effects of (B) MK-2206 or (C) Torin-1 on contralateral hypersensitivity to mechanical (left) and thermal (right) stimuli. Data are expressed as mean ± SEM (*n* = 8-10 per group); two-way ANOVA with post hoc Šídák's test. *$P<0.05$, **$P<0.01$, ***$P<0.001$ compared to Form-SD. (D, E) (Related to Figure 4B) Densitometry quantification of (D) LC3B and (E) ATG5 protein levels, normalized to β-actin. Data are expressed as mean ± SEM (*n* = 4 per group); one-way ANOVA with post hoc Šídák's test. (F) (Related to Figure 4C, D) Representative immunofluorescent images for LC3B (red) and neuronal marker NeuN (green) in L4-L6 spinal cord of vehicle- or formalin-injected mice fed either SD or MD-1. Nuclei are stained with DAPI. Magnification: 10x. Scale bar: 100 µm.

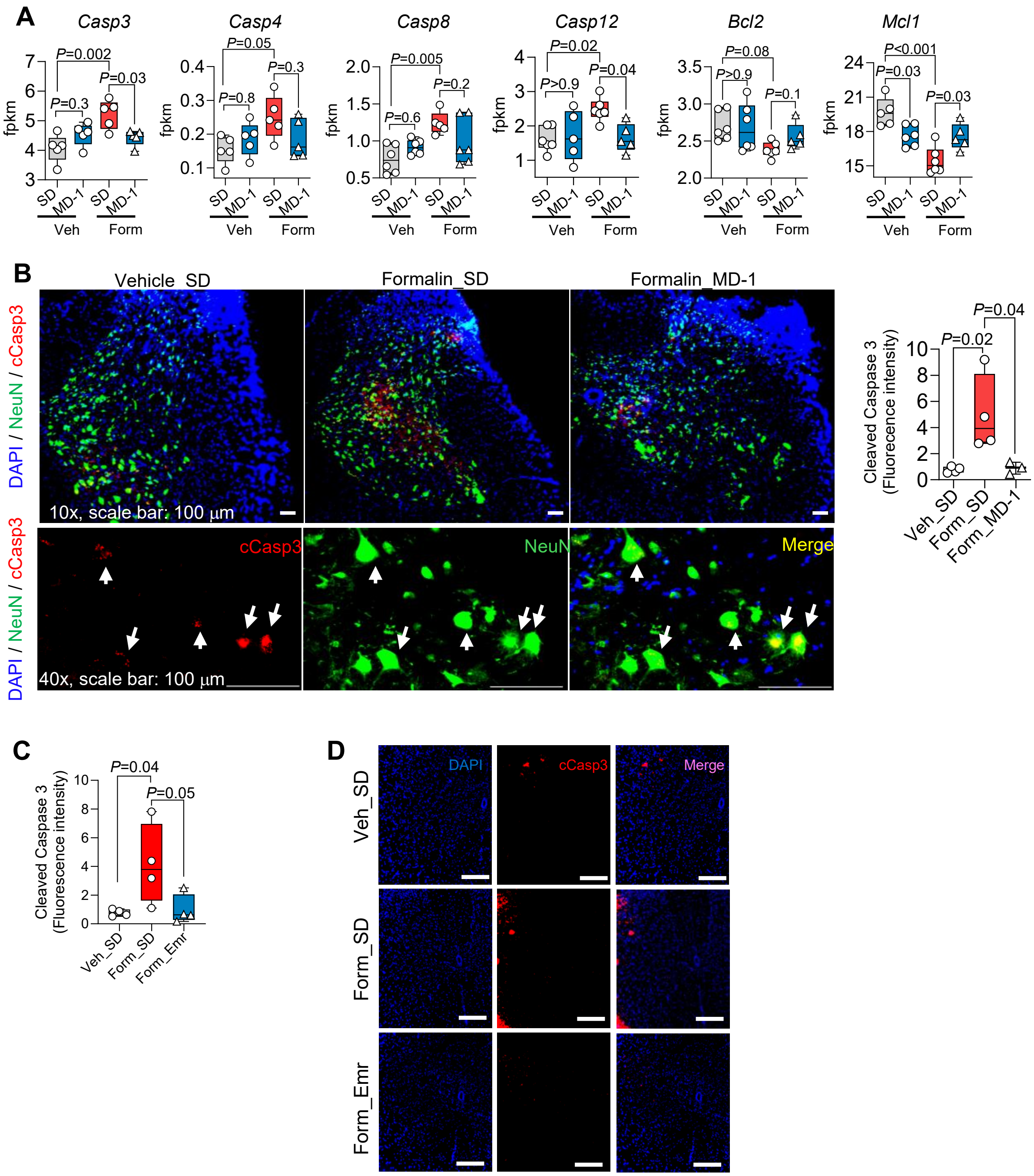

Figure S6. MD-1 prevents formalin-induced apoptosis in L4-L6 spinal cord tissue. (A, B) (Related to Figure 4) Formalin injection triggers apoptosis in L4-L6 spinal cord and MD-1 prevents this response. (A) Transcription of proapoptotic (*Casp3*, *Casp4*, *Casp8*, *Casp12*) and survival (*Bcl2*, *Mcl1*) genes in vehicle (Veh)- or formalin (Form)-injected mice fed SD or MD-1. Boxplots show individual and mean ± SEM data for vehicle-injected mice fed SD (gray boxes) and formalin-injected mice fed SD (red boxes) or MD-1 (blue boxes) (n = 5 per group); one-way ANOVA followed by Šídák's test. (B) Immunofluorescence localization of caspase-3 in L4-L6 spinal cord. Top: representative images (10x magnification) showing caspase-3 (red) and NeuN (green) immunoreactivity. Nuclei are counterstained with DAPI (blue). Scale bar, 100 µm. Bottom row: images (40x magnification) highlighting the colocalization of activated caspase-3 with NeuN. Right panel: Quantification of activated caspase-3 immunofluorescence. Statistical significance was determined by one-way ANOVA with post hoc Šídák's test. (C, D) (Related to Figure 4) Caspase inhibitor emricasan (Emr, 3 mg/kg, IP) or its vehicle (Veh) was administered to SD-fed mice on days 2-4 following intraplantar injection of formalin (1%, v/v). (C) Quantification of cleaved caspase-3 (cCasp3) immunofluorescence in the L4-L6 spinal cord of vehicle (Veh)- or formalin (Form)- injected mice fed SD. (D) Representative immunofluorescent images for cCasp3 (red) in the L4-L6 spinal cord tissues. Nuclei are stained with DAPI. Magnification: 10x. Scale bar: 100 µm. Data are expressed as mean ± SEM and analyzed by two-way ANOVA followed by Dunnett's test.

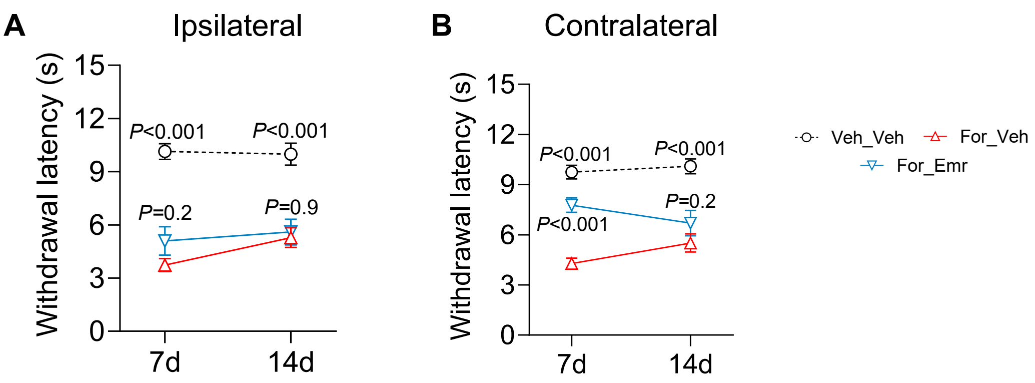

Figure S7. (Related to Figure 4) Formalin-induced pain hypersensitivity is not prevented by caspase inhibition. (A-B) Caspase inhibitor emricasan (3 mg/kg, IP) or its vehicle (Veh) was administered to SD-fed mice on days 2-4 following intraplantar injection of formalin (1%, v/v). Thermal nociceptive thresholds were assessed in the ipsilateral (A) and contralateral (B) hind paws on days 7 and 14. Data are presented as mean ± SEM (n = 7–8 mice per group). Statistical significance was determined using two-way ANOVA followed by Dunnett's multiple comparisons test. $P < 0.05$ compared to formalin-injected SD-fed mice (Form_SD).

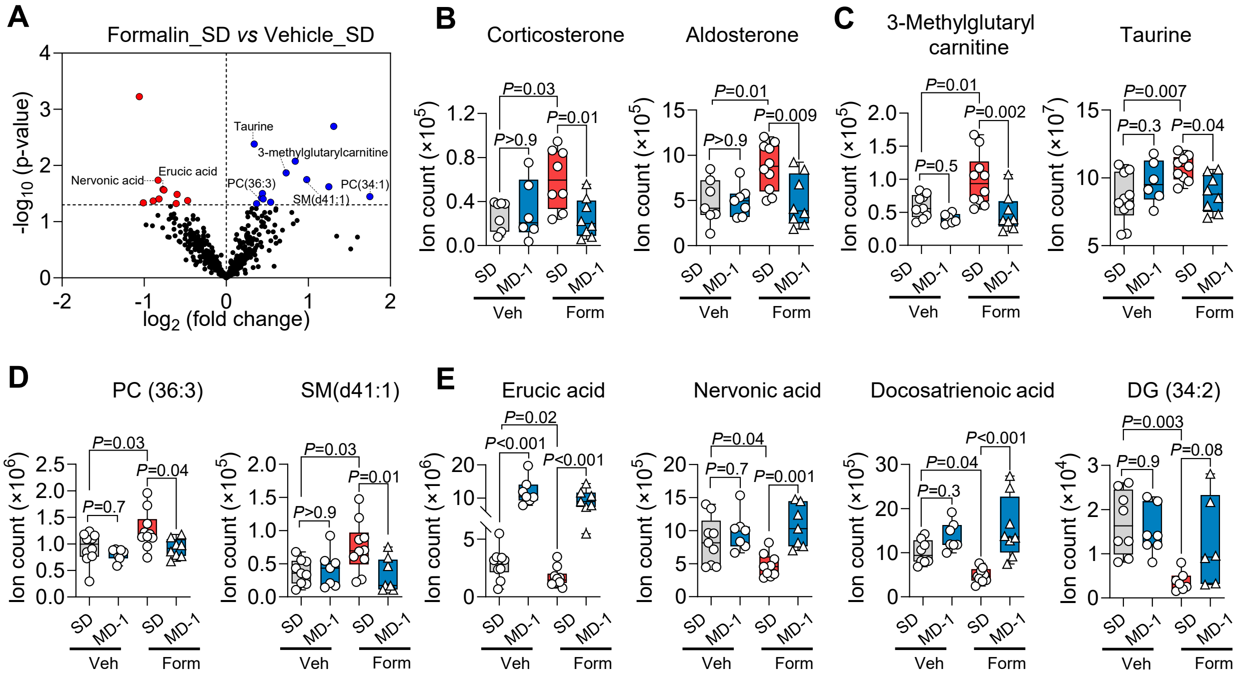

Figure S8. (Related to Figures 1, 2, and 3) Effects of hind-paw formalin injection and MD-1 exposure on the circulating metabolome. (A) Volcano plot showing metabolite changes in serum of SD-fed mice. Red dots: downregulated metabolites; blue dots: upregulated metabolites; black dots: metabolites with no significant change ($P > 0.05$) in formalin-injected SD-fed mice compared to vehicle-injected SD-fed controls. (B-E) Boxplots showing individual and mean ± SEM metabolite content (ion counts) in serum of formalin (Form)- or vehicle (Veh)-injected mice fed SD or MD-1 (n = 7-10 per group); one-way ANOVA followed by Šídák's multiple comparisons test.

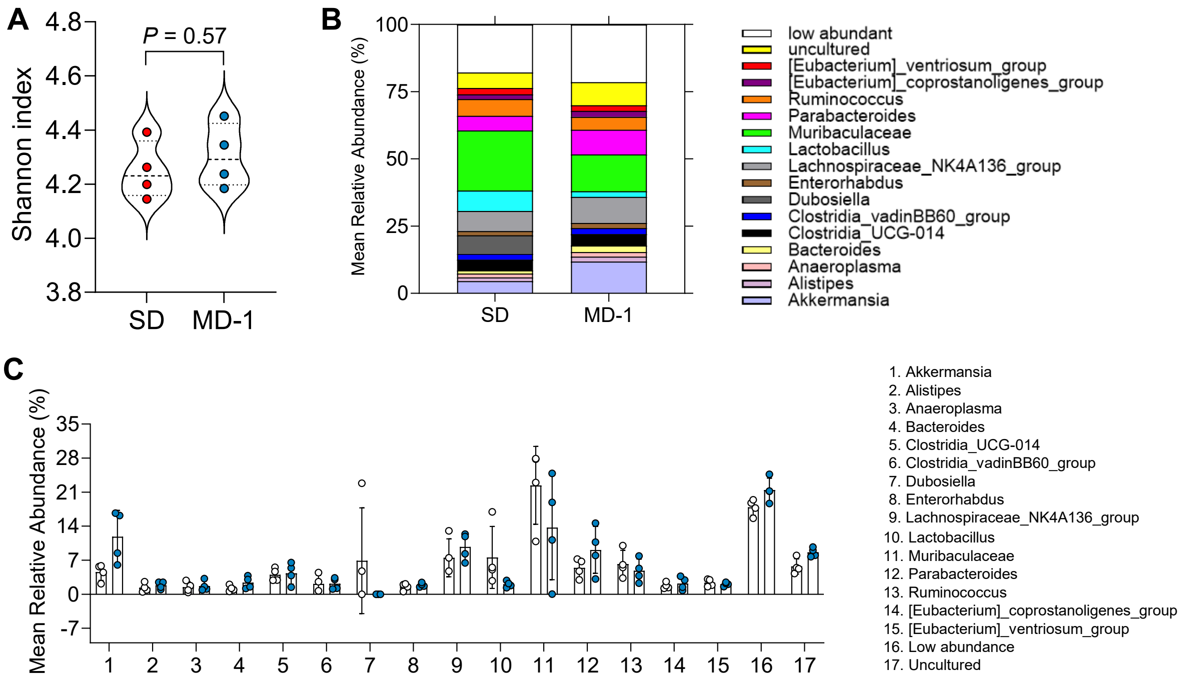

Figure S9. (Related to Figures 1, 2, and 3) MD-1 administration does not alter intestinal microbiome diversity or composition. (A) Shannon diversity index of fecal samples (*n* = 4 cages) from vehicle-injected mice fed SD or MD-1 for 25 days. (B) Relative abundance of predominant intestinal bacterial genera between groups. (C) Intestinal microbiome composition, showing bacterial genera that constitute >1% of total microbiome community.

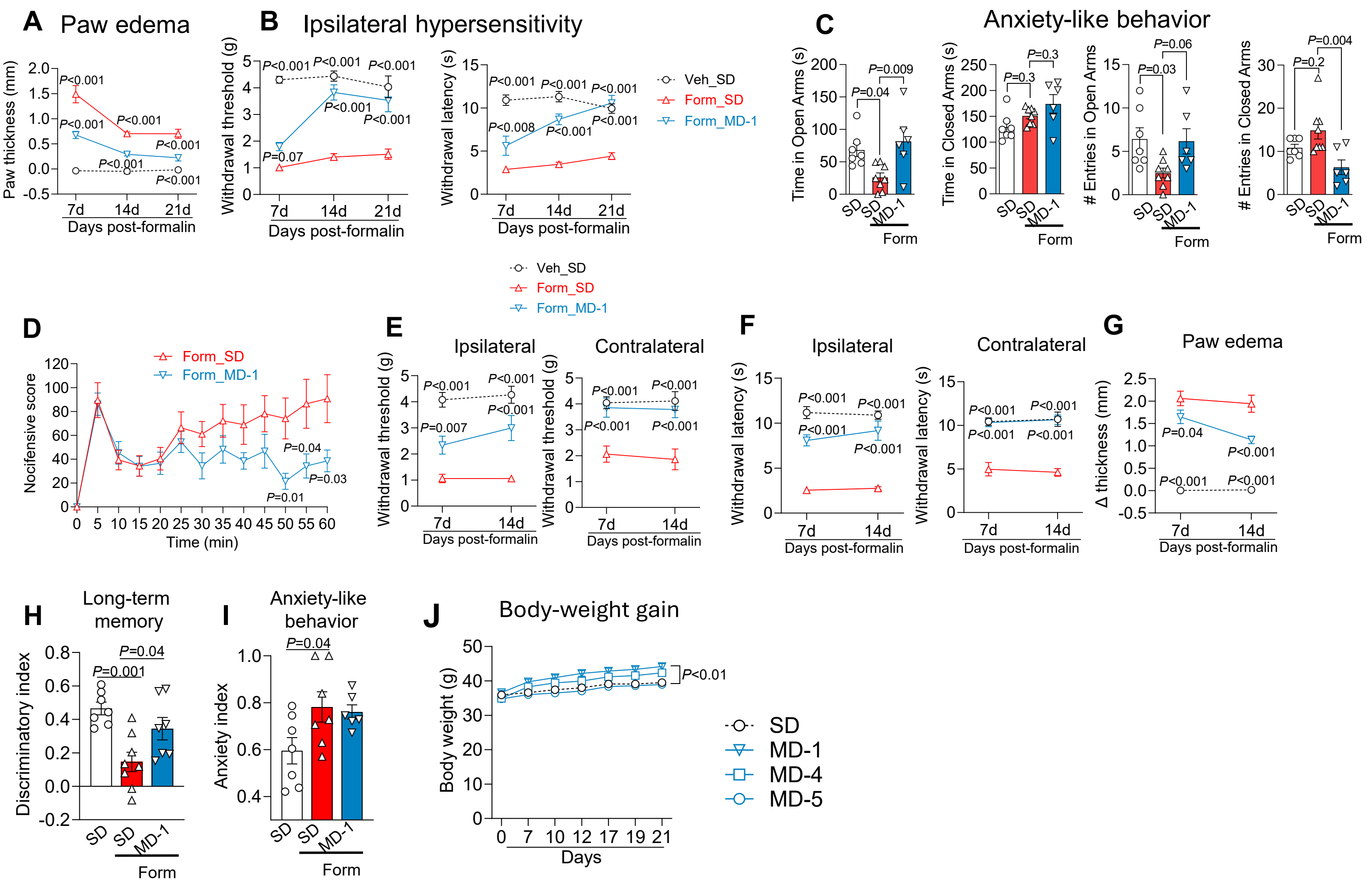

Figure S10. (Related to Figure 5) Effects of MD-1 in formalin-injected mice fed SD (red symbols) or MD-1 (blue symbols). Open circles indicate vehicle-injected mice. (A, B) Time-course of (A) paw edema; and (B) ipsilateral hypersensitivity to mechanical (left) and thermal (right) stimuli. (C) Anxiety-like behavior (elevated plus maze). Data are expressed as mean ± SEM (n = 6-10 per group); one-way ANOVA with post hoc Šídák's test; **P*<0.05, ***P*<0.01, ****P*<0.001 compared to formalin/SD. (D-I) Effects of MD-1 in female mice fed SD (red symbols) or MD-1 (blue symbols). (D) Acute nocifensive response to formalin. (E-G) Time-course of bilateral hypersensitivity to (E) mechanical and (F) thermal stimuli, and (G) paw edema. (H) Long-term memory (24-hour novel object recognition). (I) Anxiety-like behavior (elevated plus maze). Data are expressed as mean ± SEM (*n* = 5-10 per group); one-way ANOVA with post hoc Šídák's test; **P*<0.05, ***P*<0.01, ****P*<0.001 compared to formalin/SD. (J) Body-weight trajectory in male mice fed SD, MD-1, MD-4 or MD-5. Data are expressed as mean ± SEM (*n* = 8-10 per group); two-way ANOVA followed by post hoc Dunnett's test. *P*<0.01 MD vs. SD.

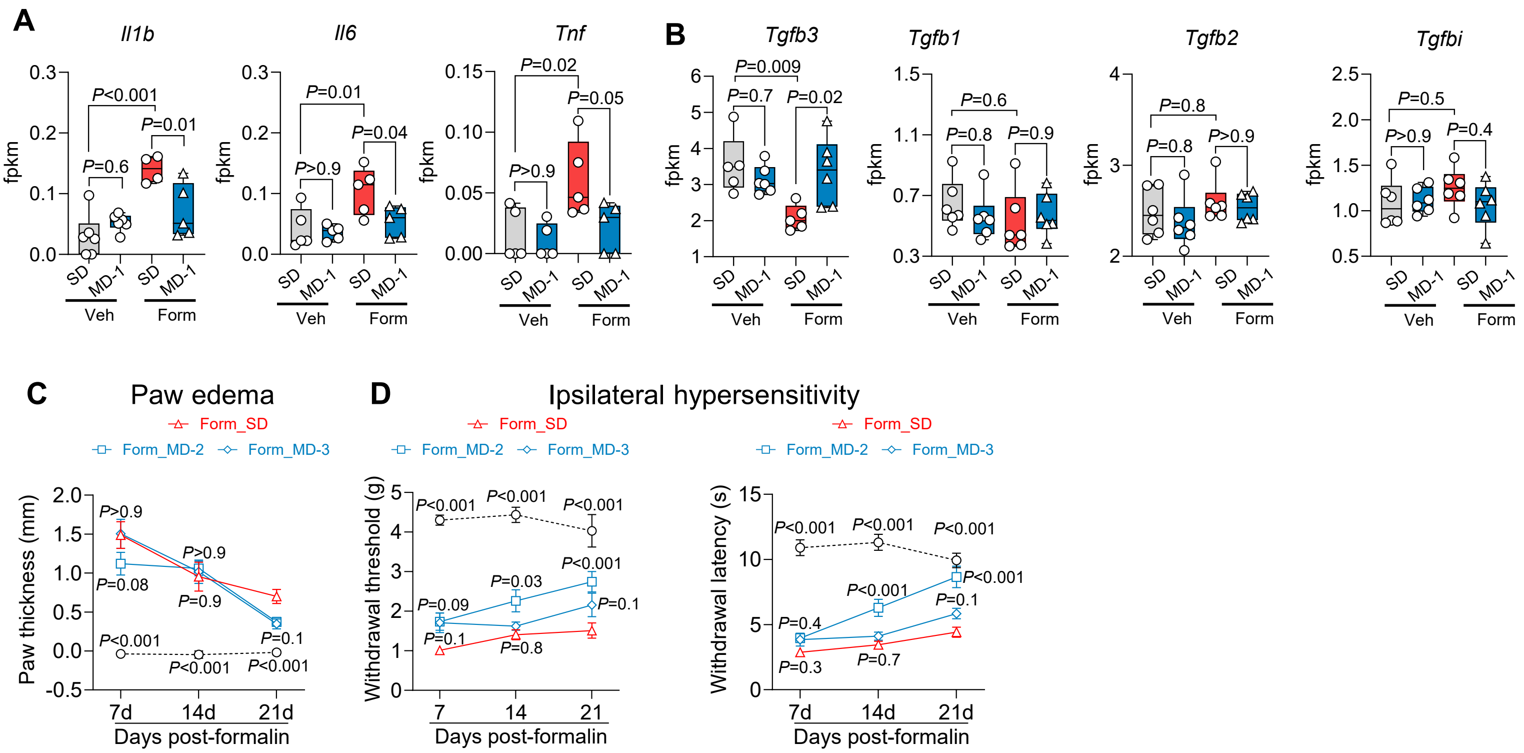

Figure S11. MD-1 modulates inflammatory and reparative gene expression in L4–L6 spinal cord, and MD-2 and MD-3 attenuate pain and edema responses in formalin-injected mice. (Related to Figure 5) (A, B) Transcription of (A) proinflammatory and (B) tissue-reparative genes in ipsilateral L4-L6 spinal cord of formalin- or vehicle-injected mice fed SD or MD-1. Boxplots show individual and mean ± SEM data for vehicle-injected mice fed SD (gray boxes) and formalin-injected mice fed SD (red boxes) or MD-1 (blue boxes) (n = 4-6 per group); one-way ANOVA followed by Šídák's test. (C, D) Effects of SD (red triangles), MD-2 (blue squares), or MD-3 (blue diamonds) in formalin-injected mice. Open circles indicate vehicle-injected mice fed SD. Time-course of (C) paw edema and (D) ipsilateral hypersensitivity to mechanical (left) and thermal (right) stimuli. Data are expressed as mean ± SEM (n = 8-10 per group); one-way ANOVA followed by Šídák's test. *$P<0.05$, **$P<0.01$, ***$P<0.001$ compared to formalin/SD.

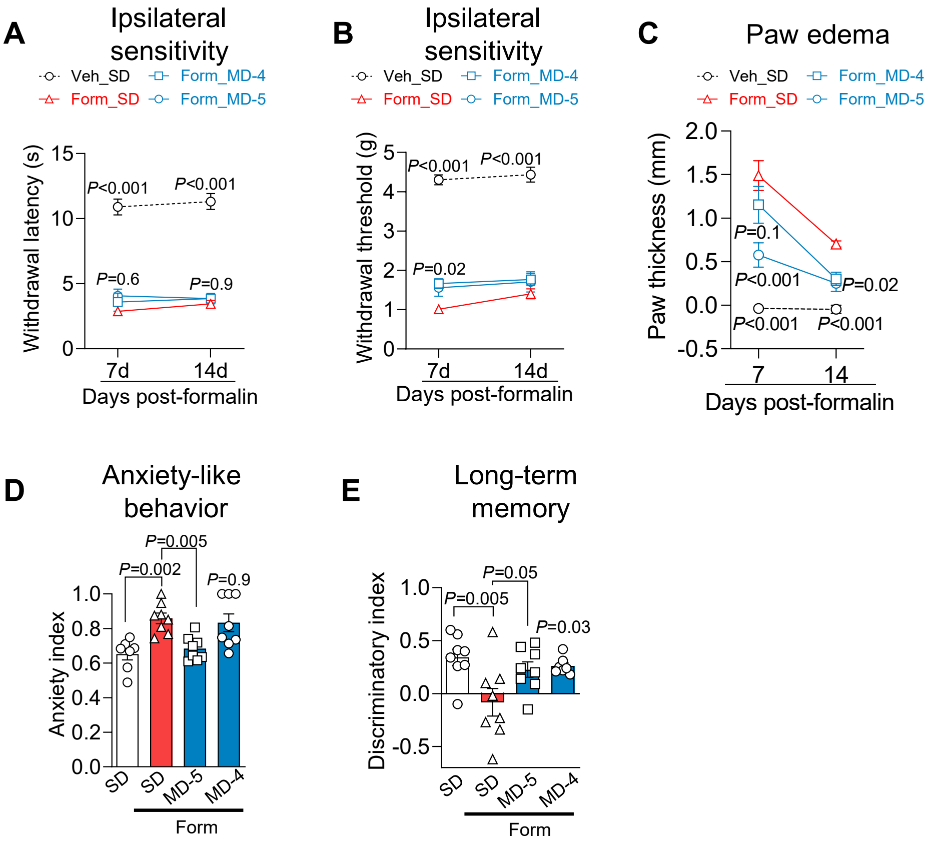

Figure S12. (Related to Figure 5J-K) Effects of MD-4 and MD-5 in formalin-injected mice fed SD (red triangles), MD-4 (blue squares), or MD-5 (blue circle). (A-C) Time-course of (A, B) ipsilateral hypersensitivity to (A) thermal and (B) mechanical stimuli and (C) paw edema. (D) Anxiety-like behavior (elevated plus maze). (E) Long-term memory (24-hour novel object recognition). Data are expressed as mean ± SEM (n = 8-10 per group); one- or two-way ANOVA followed by Šídák's test. **P*<0.05, ***P*<0.01, ****P*<0.001 compared to formalin/SD.

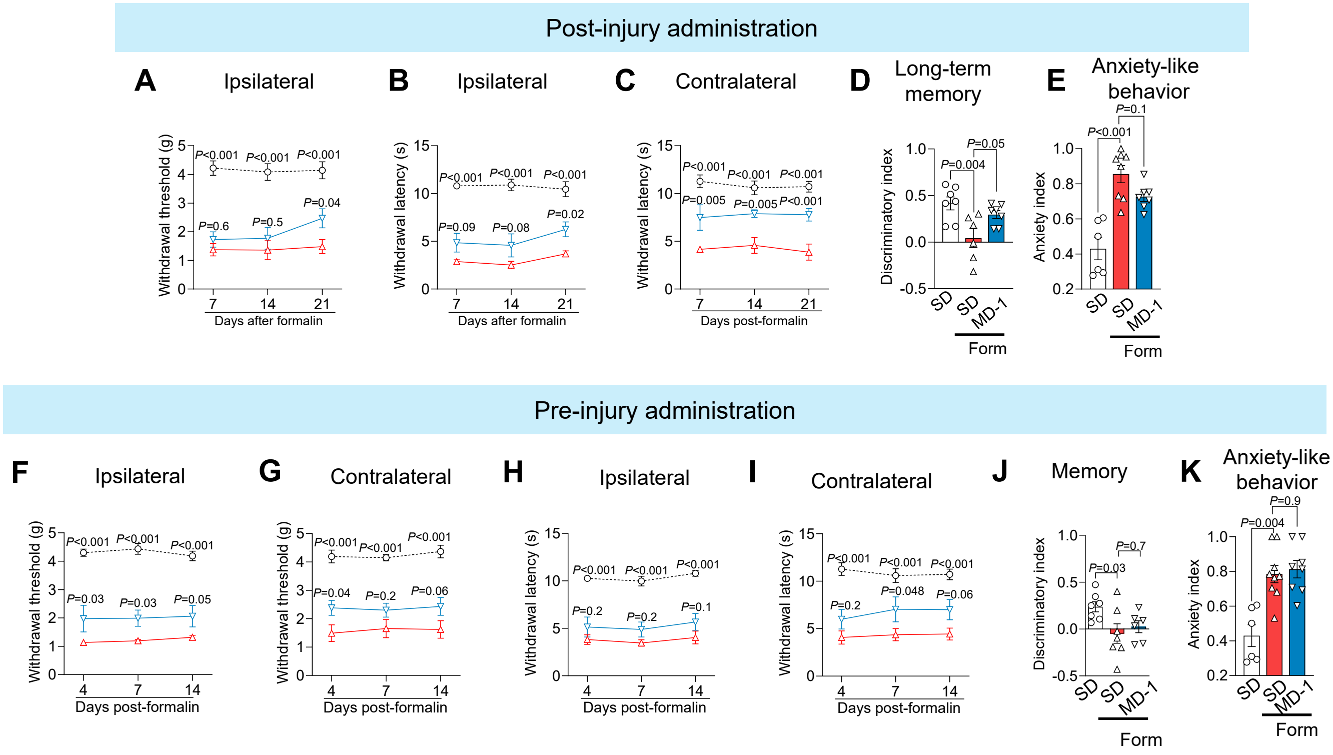

Figure S13. (Related to Figure 5L, M) Effects of timing of MD-1 exposure in formalin-injected mice fed SD (red symbols) or MD-1 (blue symbols). Open circles indicate vehicle-injected mice fed SD. (A-C) Time-course of the effects of post-injury MD-1 administration on (A, B) ipsilateral hypersensitivity to (A) mechanical and (B) thermal stimuli; and (C) contralateral thermal hypersensitivity. (D) Effects of post-injury MD-1 administration on long-term memory (24-hour novel object recognition). (E) Effects of post-injury MD-1 administration on anxiety-like behavior (elevated plus maze). (F-K) Time-course of the effects of pre-injury MD-1 administration on (F) ipsilateral mechanical hypersensitivity, (G) contralateral mechanical hypersensitivity, (H) ipsilateral thermal hypersensitivity, (I) contralateral thermal hypersensitivity. (J) Effects of pre-injury MD-1 administration on long-term memory (24-hour novel object recognition). (K) Effects of pre-injury MD-1 administration on anxiety-like behavior (elevated plus maze).. Data are expressed as mean ± SEM (n = 8-10 per group); one- or two-way ANOVA with Šídák's test. *P<0.05, **P<0.01, ***P<0.001 compared to formalin/SD.

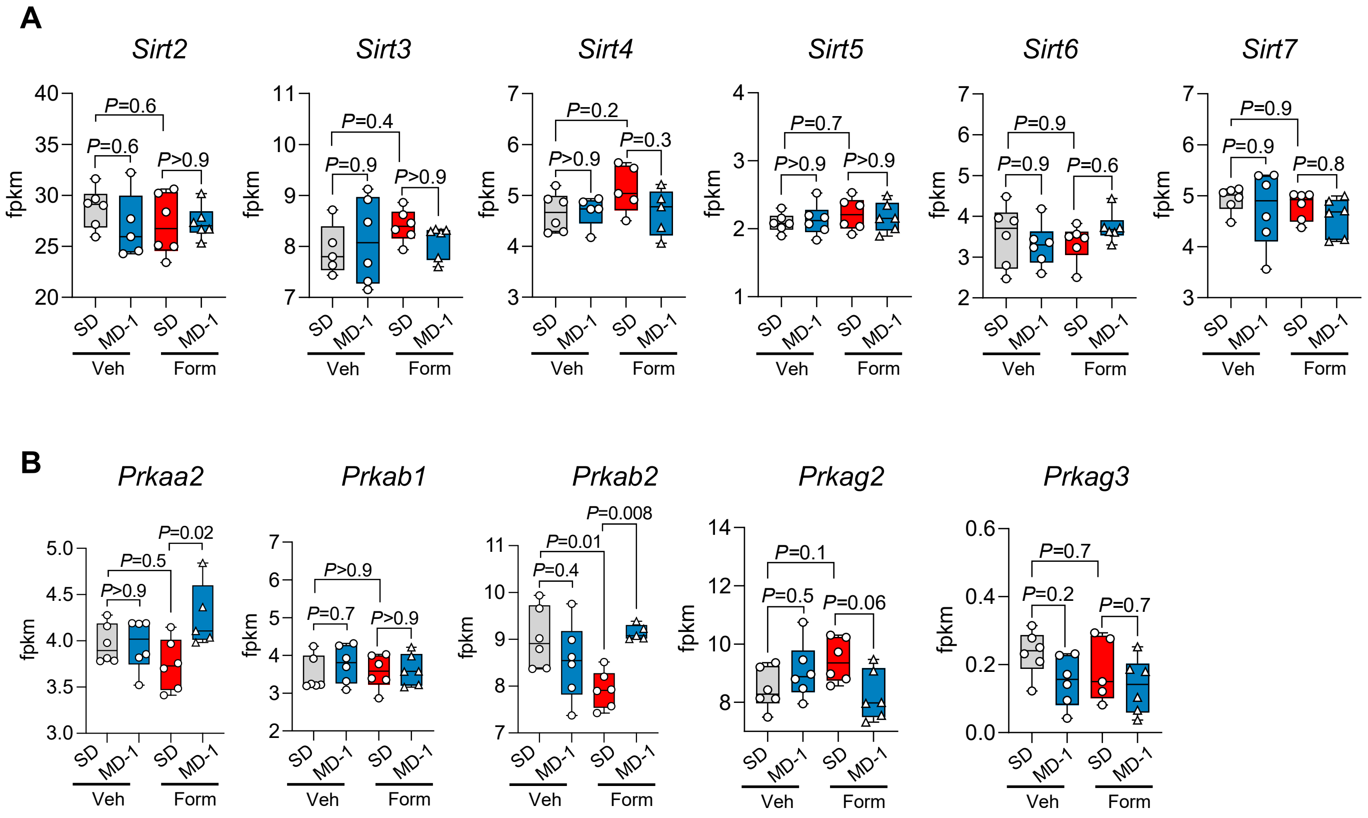

Figure S14. MD-1 modulates transcription of sirtuin family members and AMPK subunits in the ipsilateral L4–L6 spinal cord after formalin injection. (Related to Figure 6) (A) Transcription of sirtuin family members (*Sirt2*-*Sirt7*) in ipsilateral L4-L6 spinal cord of vehicle (Veh)- or formalin (Form)-injected mice fed SD or MD-1. Boxplots show individual and mean ± SEM data for vehicle-injected mice fed SD (gray boxes) and formalin-injected mice fed SD (red boxes) or MD-1 (blue boxes). Data are expressed as mean ± SEM (*n* = 5-6 per group); one-way ANOVA with post hoc Šídák's test. (B) Transcription of AMPK subunits in ipsilateral L4-L6 spinal cord of vehicle (Veh)- or formalin (Form)-injected mice fed SD or MD-1. Boxplots show individual and mean ± SEM data for vehicle-injected mice fed SD (gray boxes) and formalin-injected mice fed SD (red boxes) or MD-1 (blue boxes). Data are expressed as mean ± SEM (*n* = 5-6 per group); one-way ANOVA with post hoc Šídák's test.

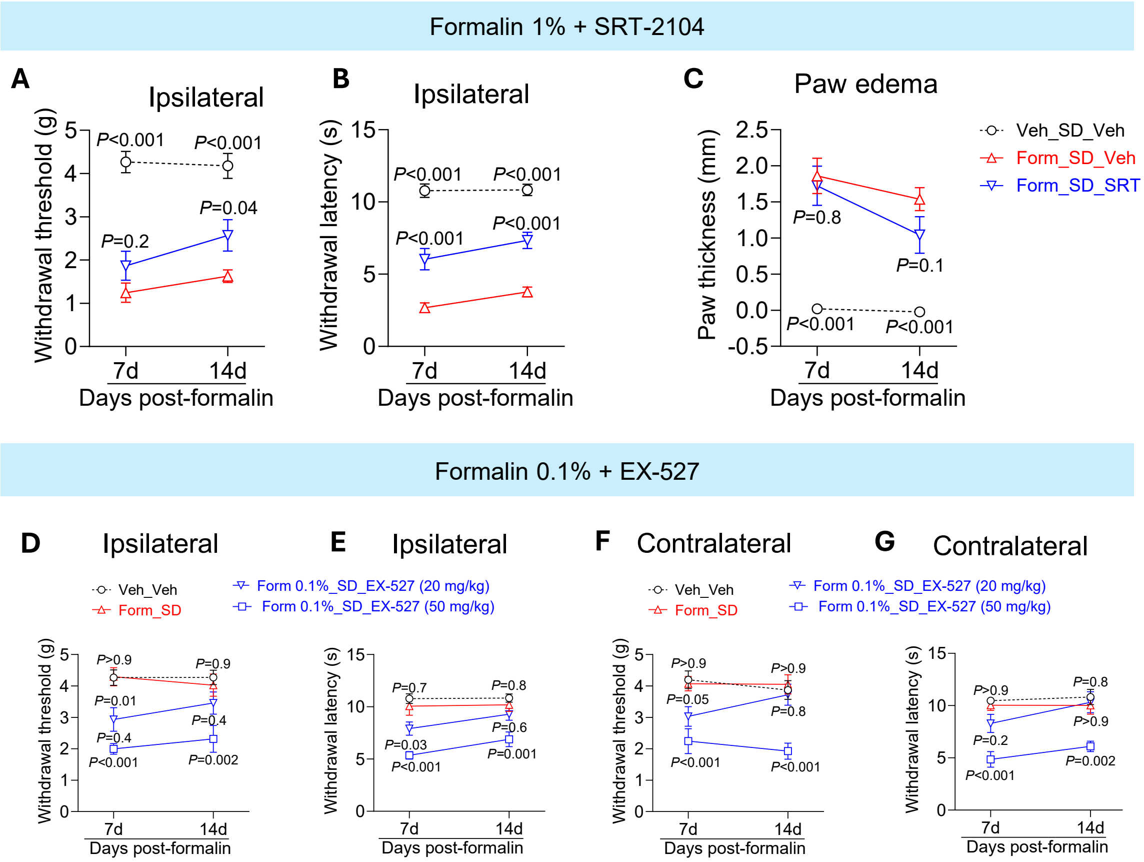

Figure S15. Pharmacological modulation of SIRT1 alters formalin-induced pain and edema. (Related to Figure 6I-K) (A-C) Effects of SIRT1 activator SRT-2104 (100 mg/kg, IP) on ipsilateral hypersensitivity to (A) mechanical and (B) thermal stimuli and (C) paw edema in formalin-injected mice fed SD (red symbols) or MD-1 (blue symbols). Open circles indicate vehicle-injected mice fed SD. (D-G) Effects of SIRT1 inhibitor EX-527. EX-527 (20 and 50 mg/kg, IP) or its vehicle (Veh) was administered to SD-fed mice on days 2-4 following intraplantar injection of formalin (0.1%, v/v). Hypersensitivity to mechanical (D, F) and thermal (E, G) stimuli were assessed in the ipsilateral and contralateral hind paws on days 7 and 14. Data are presented as mean ± SEM (n = 7–8 mice per group). Statistical significance was determined using two-way ANOVA followed by Šídák's test. P < 0.05 compared to formalin-injected SD-fed mice (Form_SD).

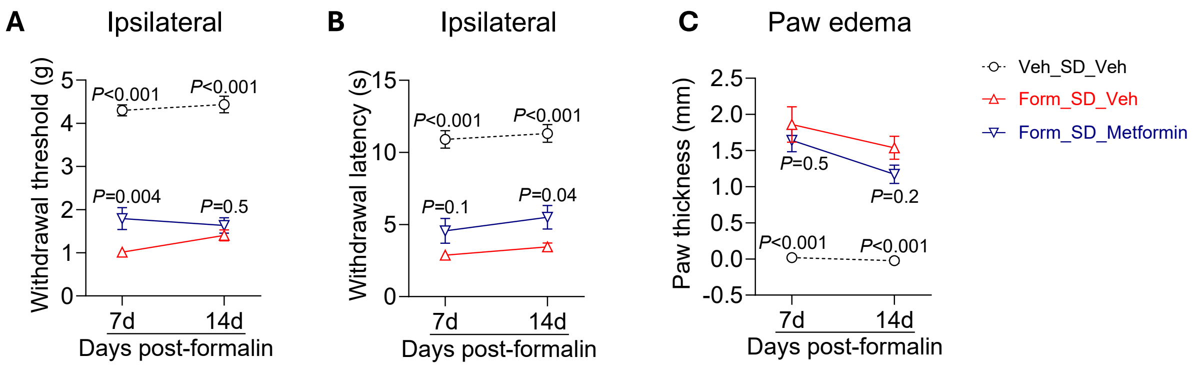

Figure S16. (Related to Figure 6). Activation of AMPK alleviates formalin-induced pain hypersensitivity. (A-C) Effects of AMPK activator metformin (200 mg/kg, IP) on ipsilateral hypersensitivity to (A) mechanical and (B) thermal stimuli and (C) paw edema. Data are expressed as mean ± SEM (*n* = 7-10 per group); two-way ANOVA with Šídák's test; **P*<0.05, ***P*<0.01, ****P*<0.001 vs. formalin/SD.

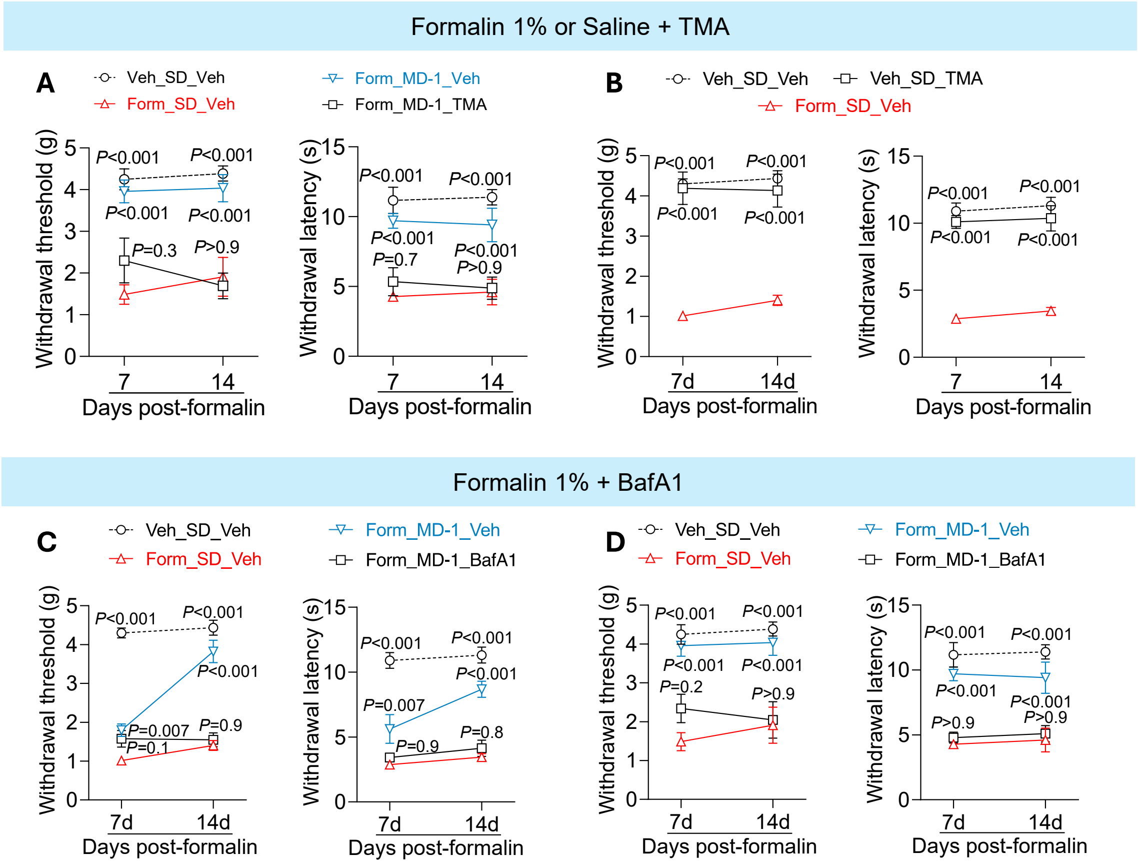

Figure S17. (Related to Figure 6) Effects of autophagy inhibition on the response to MD-1. (A) Treatment with autophagy inhibitor TMA (30 mg/kg, IP) on days 2-4 after formalin (1% vol/vol) negated the protective effects of MD-1 on formalin-induced mechanical (left) and thermal (right) hypersensitivity in the contralateral paws. (B) Effects of TMA on ipsilateral mechanical (left) and thermal (right) hypersensitivity in saline-injected mice. (C-D) Post-injury administration of autophagy inhibitor bafilomycin A1 (BafA1, 1 mg/kg, IP) negated the protective effects of MD-1 on formalin-induced ipsilateral (C) and contralateral (D) hypersensitivity to mechanical (left) and thermal (right) stimuli. Open circles: vehicle (veh)/SD/veh; red triangles: formalin/SD/veh; blue triangles: formalin/MD-1/veh; open squares, formalin/MD-1/TMA or BafA1. Statistical significance was determined by one- or two-way ANOVA followed by Dunnett or Šídák multiple comparisons test. *P<0.05, **P<0.01, ***P<0.001 compared to formalin-SD (n = 7-10).

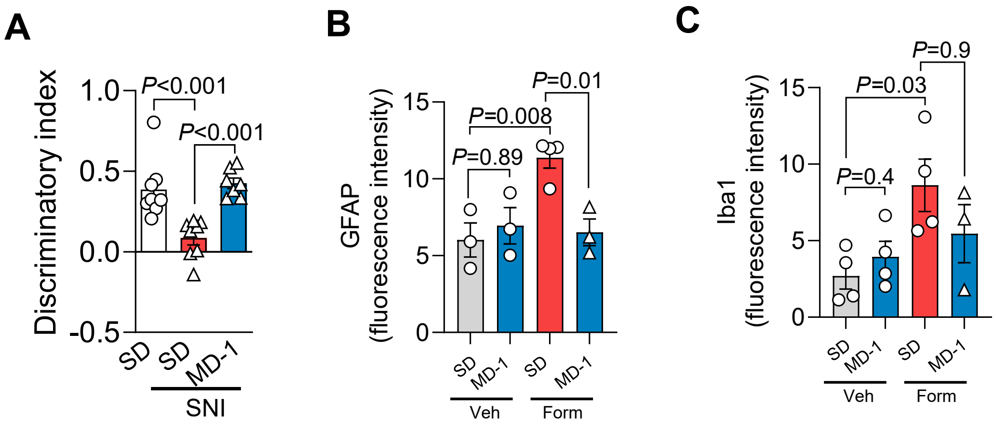

Figure S18. (Related to Figure 7A-E). MD-1 mitigates SNI-induced cognitive deficits and modulates spinal glial activation after formalin injection. (A) Effects of MD-1 administration on SNI-induced long-term memory deficits (24-hour novel-object recognition). The test was performed on post-SNI day 21. Data are expressed as mean ± SEM (n = 7-8 per group); one-way ANOVA with Šídák's test; **P*<0.05, ***P*<0.01, ****P*<0.001 vs. SNI-SD group. (B) Quantification of GFAP immunofluorescence in the L4-L6 spinal cord of vehicle (Veh)- or formalin (Form)-injected mice fed SD or MD-1. Data are mean ± SEM and analyzed by two-way ANOVA followed by Dunnett's test. (C) Quantification of IBA-1 immunofluorescence in the L4-L6 spinal cord of vehicle (Veh)- or formalin (Form)-injected mice fed SD or MD-1. Data are mean ± SEM and analyzed by two-way ANOVA followed by Dunnett's test.

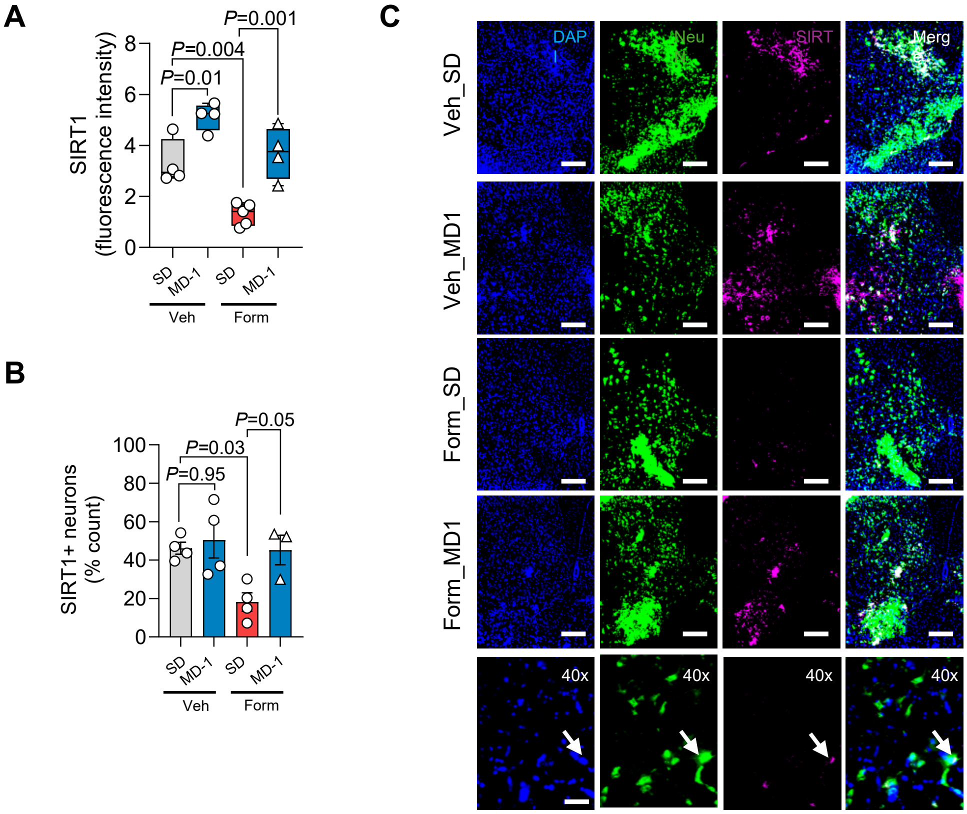

Figure S19. (Related to Figure 7A-E) MD-1 enhances spinal SIRT1 expression following formalin injection. (A-B) Quantification of SIRT1 immunofluorescence in the L4-L6 spinal cord of vehicle (Veh)- or formalin (Form)- injected mice fed SD or MD-1. (C) Representative immunofluorescent images for SIRT1 (magenta) and neuronal marker NeuN (green) in the L4-L6 spinal cord tissues. Nuclei are stained with DAPI. Magnification: 10x. Scale bar: 100 µm. Arrows indicate SIRT1 and NeuN colocalization at 40x. Data are mean ± SEM and analyzed by two-way ANOVA followed by Dunnett's test.

A.

Ingredient	SD	MD-1	MD-2	MD-3	MD-4	MD-5
Ala	1.20	2.80	2.00	1.60	2.80	1.20
Thr	0.60	1.76	1.18	0.89	1.76	0.60
Pro	1.90	3.84	2.87	2.39	3.84	1.90
Ser	0.90	1.68	1.29	1.10	1.68	0.90
Leu	2.30	4.85	3.58	2.94	4.85	2.30
Ile	0.70	2.98	1.84	1.27	2.98	0.70
Val	0.90	2.98	1.94	1.42	2.98	0.90
Phe	1.00	2.26	1.63	1.32	2.26	1.00
Tyr	0.50	1.28	0.89	0.70	1.28	0.50
Met	0.50	0.98	0.74	0.62	0.98	0.50
Cys	0.30	0.68	0.49	0.40	0.68	0.30
Trp	0.20	0.76	0.48	0.34	0.76	0.20
Oleic acid	1.10	6.06	3.58	2.34	6.06	1.10
Erucic acid	0.00	0.25	0.13	0.06	0.25	0.00
PEA	0.00	0.08	0.04	0.02	0.00	0.08

B.

	Carbohydrate	Fat	Protein	Energy density	Daily intake (g/100g)
SD	47.0	6.5	19.1	3.2	13.5 ± 0.8
MD-1	35.7	10.2	33.0	3.7	11.8 ± 1.9
MD-2	41.4	9.1	28.4	3.6	ND
MD-3	44.2	7.8	23.7	3.4	ND
MD-4	35.7	10.2	33.0	3.7	ND
MD-5	47.0	6.6	19.1	3.2	ND

Supplemental Table S1. (A) Composition of standard diet (SD) and modified diets (MD) used in the present study. Data are expressed as the percentage of each component's weight relative to the total weight of the diet (% g/g total). (B) Estimated caloric content for each diet, expressed as a percentage of the diet's total calories, including the contribution of carbohydrates, proteins, and lipids to the total caloric content. The daily intake for SD and MD-1(g/100 g body weight) is also provided (*n* = 4). Abbreviations: ND, Not Determined.

	Veh_SD		Veh_MD-1			
**Compound Id**	**Mean**	**SEM**	**Mean**	**SEM**	**Log_2 (FC)**	***P*-value**
Methionine sulfoxide	3.87E+03	8.83E+02	8.62E+02	1.14E+02	-2.17	7.78E-03
Glutarylcarnitine	5.12E+05	6.26E+04	2.76E+05	3.53E+04	-0.89	5.71E-03
Saccharopine	3.66E+06	4.28E+05	2.01E+06	3.21E+05	-0.87	7.40E-03
N2-Acetylornithine	4.03E+03	3.38E+02	2.31E+03	5.67E+02	-0.80	2.60E-02
Proline betaine	1.18E+05	7.84E+03	7.30E+04	7.45E+03	-0.69	9.38E-04
PS(18:0/18:2(9Z,12Z))	2.22E+06	2.13E+05	1.39E+06	1.00E+05	-0.67	3.88E-03
DL-2-Aminooctanoic acid	1.10E+05	5.12E+03	6.96E+04	5.14E+03	-0.67	5.66E-05
LysoPE(0:0/20:2(11Z,14Z))	4.02E+04	3.34E+03	2.73E+04	3.35E+03	-0.56	1.65E-02
(10Z,12Z)-octadeca-10,12-dienoylcarnitine	4.79E+05	1.99E+04	3.26E+05	2.50E+04	-0.55	3.88E-04
L-Pipecolic acid	4.22E+04	1.18E+03	2.88E+04	1.26E+03	-0.55	2.04E-06
3,4-Dihydroxyhydrocinnamic acid	1.42E+06	1.33E+05	9.90E+05	1.08E+05	-0.52	2.52E-02
D-Glucuronic acid	7.82E+03	3.99E+02	5.49E+03	5.94E+02	-0.51	7.41E-03
PE(18:0/18:3(9Z,12Z,15Z))	3.05E+05	2.05E+04	2.14E+05	1.36E+04	-0.51	2.27E-03
Taurodeoxycholic acid	1.68E+04	1.72E+03	1.19E+04	1.35E+03	-0.50	4.08E-02
Isolinderanolide	2.17E+06	1.35E+05	1.56E+06	1.17E+05	-0.48	3.47E-03
4-Hydroxyproline	2.71E+05	2.03E+04	2.00E+05	1.86E+04	-0.44	2.17E-02
(Ã‚Â±)-2-Hydroxy-4-(methylthio)butanoic acid	1.56E+04	1.09E+03	1.16E+04	1.01E+03	-0.43	1.63E-02
Allantoin	8.62E+05	4.91E+04	6.47E+05	3.12E+04	-0.41	2.38E-03
Stoloniferone c isomer	7.10E+05	5.60E+04	5.44E+05	3.14E+04	-0.38	2.26E-02
LysoPE(20:1(11Z)/0:0)	2.96E+06	1.82E+05	2.38E+06	1.89E+05	-0.32	4.29E-02
Normetanephrine	8.02E+03	5.12E+02	6.46E+03	4.92E+02	-0.31	4.43E-02
L-Fucose	1.00E+05	7.60E+03	8.11E+04	3.89E+03	-0.31	4.18E-02
Pantothenic acid	2.41E+05	8.26E+03	1.96E+05	1.46E+04	-0.30	2.30E-02

Saringosterol isomer	4.04E +06	1.97E +05	3.30E +06	2.20E +05	-0.29	2.68E -02
Uridine diphosphate glucuronic acid	9.93E +04	3.54E +03	8.13E +04	4.74E +03	-0.29	1.02E -02
(2R,3R,4R)-2-Amino-4-hydroxy-3-methylpentanoic acid	4.16E +05	2.12E +04	3.41E +05	1.88E +04	-0.28	1.91E -02
LysoPC(20:2(11Z,14Z)/0:0)	1.56E +05	6.07E +03	1.29E +05	6.25E +03	-0.27	8.87E -03
Uridine diphosphate-N-acetylgalactosamine	3.22E +06	9.54E +04	2.84E +06	1.29E +05	-0.18	3.80E -02
N-Acetyl-1-aspartylglutamic acid	2.14E +08	6.50E +06	1.91E +08	6.66E +06	-0.16	2.65E -02
PE(20:4(6E,8Z,11Z,14Z)+=O(5)/P-18:1(9Z))	2.34E +06	8.05E +04	2.68E +06	1.09E +05	0.20	2.72E -02
Acetylcholine	6.14E +07	2.38E +06	7.05E +07	3.27E +06	0.20	4.32E -02
PE(41:7)	3.75E +05	2.59E +04	4.41E +05	1.17E +04	0.23	3.95E -02
PE(18:0/18:1(9Z))	7.36E +05	3.38E +04	8.67E +05	3.07E +04	0.24	1.17E -02
PE(16:1(9Z)/22:0)	7.03E +05	3.05E +04	8.43E +05	2.90E +04	0.26	4.77E -03
2-Arachidonoyl glycerol	5.19E +06	2.01E +05	6.27E +06	4.05E +05	0.27	4.13E -02
PE(20:4(8Z,11Z,14Z,17Z)/22:6(4Z,7Z,10Z,13Z,16Z,19Z))	1.06E +05	7.78E +03	1.29E +05	5.71E +03	0.28	3.23E -02
2-Methylglutaric acid	2.71E +04	1.10E +03	3.31E +04	1.65E +03	0.29	1.20E -02
Butyric acid	1.95E +04	1.11E +03	2.38E +04	7.97E +02	0.29	6.87E -03
SM(d18:1/18:0)	4.22E +06	3.48E +05	5.28E +06	2.16E +05	0.33	2.05E -02
trans-Aconitic acid	2.32E +06	1.62E +05	3.01E +06	1.61E +05	0.38	8.56E -03
PC(22:5(4Z,7Z,10Z,13Z,16Z)/16:0)	1.11E +06	7.14E +04	1.46E +06	1.08E +05	0.40	1.91E -02
N-Acetylglutamine	2.15E +06	1.02E +05	2.96E +06	2.70E +05	0.46	2.33E -02
PC(16:0/15:0)	4.78E +04	5.67E +03	6.62E +04	4.85E +03	0.47	2.64E -02
Guanidinosuccinic acid	3.22E +03	2.19E +02	4.60E +03	4.17E +02	0.51	1.66E -02
3-Hydroxybutyric acid	1.60E +05	8.45E +03	2.31E +05	2.22E +04	0.52	1.89E -02
Adenine	1.25E +08	1.49E +07	1.87E +08	2.24E +07	0.59	3.99E -02
PE(22:2(13Z,16Z)/20:4(8Z,11Z,14Z,17Z))	1.77E +05	2.36E +04	2.69E +05	1.55E +04	0.60	5.49E -03
Tiglylcarnitine	1.48E +04	1.17E +03	2.39E +04	3.65E +03	0.69	4.80E -02

Deoxycytidine	3.07E +03	4.17E +02	5.06E +03	7.56E +02	0.72	4.49E -02
Diaminopimelic acid	1.37E +03	1.74E +02	2.36E +03	2.57E +02	0.79	8.36E -03
Biotin amide	5.47E +05	3.13E +04	1.01E +06	8.86E +04	0.88	1.46E -03
(S,E)-Zearalenone	1.60E +05	9.83E +03	3.06E +05	3.21E +04	0.93	3.31E -03
Alanylproline	4.12E +03	5.71E +02	7.89E +03	1.17E +03	0.94	1.78E -02
N,N-Dimethylformamide	2.31E +06	8.78E +04	4.60E +06	4.89E +05	1.00	3.07E -03
Enterodiol	1.44E +06	9.31E +04	2.92E +06	2.99E +05	1.02	1.93E -03
Melleolide M	1.92E +05	2.50E +04	4.11E +05	6.67E +04	1.09	1.62E -02
N-alpha-Acetyl-L-citrulline	3.70E +05	1.74E +04	7.91E +05	7.20E +04	1.10	8.66E -04
L-Allothreonine	8.33E +06	4.88E +05	1.92E +07	2.06E +06	1.20	1.58E -03
L-Ergothioneine	1.55E +06	4.48E +04	3.74E +06	5.13E +05	1.27	5.23E -03
2-Hydroxybutyric acid	8.46E +03	1.06E +03	2.20E +04	3.57E +03	1.38	8.15E -03
N1-Methyl-4-pyridone-3-carboxamide	9.73E +04	1.53E +04	2.61E +05	2.13E +04	1.43	4.69E -05
Val asp isomer	2.00E +05	5.67E +03	5.56E +05	4.15E +04	1.48	1.18E -04

Table S3. Spinal cord metabolites affected by MD-1 in vehicle-injected mice, ranked in ascending order of Log2 fold change (FC). Data are expressed as mean±SEM values (ion counts). Abbreviations: PS, phosphatidylserine; Val, valine; Asp, aspartate; FC: fold changes ([Veh_MD-1]/[Veh_SD]).

	Veh_SD		Form_SD			
**Compound Id**	**Mean**	**SEM**	**Mean**	**SEM**	**Log_2 FC**	***P*-value**
DG(16:0/18:2(9Z,12Z)/0:0)	1.70E+04	2.56E+03	3.75E+03	8.34E+02	-2.18	9.79E-04
Docosatrienoic acid	1.01E+06	9.66E+04	4.85E+05	5.40E+04	-1.06	5.94E-04
Indole-3-methyl acetate	3.36E+05	7.15E+04	1.67E+05	2.28E+04	-1.01	4.62E-02
5-Nonadecyl-1,3-benzenediol	2.56E+05	4.86E+04	1.38E+05	1.80E+04	-0.89	4.28E-02
Nervonic acid	8.40E+05	1.20E+05	4.72E+05	5.96E+04	-0.83	1.82E-02
Docosadienoate (22:2n6)	6.16E+05	1.06E+05	3.48E+05	4.68E+04	-0.82	3.93E-02
Erucic acid	2.88E+06	4.13E+05	1.69E+06	2.53E+05	-0.77	2.68E-02
Arachidic acid	1.39E+06	2.03E+05	8.19E+05	1.13E+05	-0.76	2.73E-02
10Z-Pentadecenoic acid	1.41E+05	2.00E+04	9.23E+04	9.83E+03	-0.61	4.77E-02
Tetracosahexaenoic acid	1.66E+06	2.17E+05	1.10E+06	8.29E+04	-0.6	3.28E-02
PG(18:0/16:0)	4.60E+04	5.31E+03	3.33E+04	1.46E+03	-0.47	4.22E-02
Taurine	8.47E+07	5.72E+06	1.07E+08	3.13E+06	0.34	4.15E-03
Butyric acid	4.34E+04	4.60E+03	5.61E+04	3.83E+03	0.37	4.79E-02
PC(18:0/18:3(9Z,12Z,15Z))	9.29E+05	9.06E+04	1.25E+06	1.13E+05	0.43	3.77E-02
Pseudouridine	3.61E+06	3.52E+05	4.90E+06	4.28E+05	0.44	3.16E-02
Glutarylcarnitine	2.53E+04	2.08E+03	3.46E+04	3.52E+03	0.45	3.93E-02
Capryloylglycine	1.89E+05	2.31E+04	2.74E+05	3.18E+04	0.54	4.50E-02
3-methylglutarylcarnitine	5.94E+04	5.87E+03	9.89E+04	1.25E+04	0.73	1.35E-02
Aldosterone	4.87E+05	8.77E+04	8.71E+05	8.22E+04	0.84	8.42E-03
SM(d18:1/23:0)	3.78E+04	6.33E+03	7.48E+04	1.22E+04	0.98	1.78E-02
Corticosterone	2.81E+04	5.11E+03	6.68E+04	1.39E+04	1.25	2.39E-02
Indole-3-carboxilic acid-sulphate isomer	7.13E+05	1.08E+05	1.77E+06	2.47E+05	1.31	2.01E-03

PC(18:1(9Z)/16:0)	7.02E+05	2.85E+05	2.36E+06	6.46E+05	1.75	3.59E-02

Table S6. Serum metabolites influenced by formalin injection, ranked in ascending order of Log_2 fold change (FC). Data are presented as mean±SEM values (ion counts) for each metabolite. DG: diacylglycerol; PG: phosphatidylglycerol; PE: phosphatidylethanolamine; PC: phosphatidylcholine. FC: fold changes ([Form_SD]/[Veh_SD]).

Dependent Variable	Source	Type III Sum of Squares	df	Mean Square	F	Sig.	Partial Eta Squared
Thermal (Ipsilateral)	Corrected Model	554.178	6	92.363	29.654	<.001	0.813
	Intercept	2358.363	1	2358.363	757.175	<.001	0.949
	BW	10.658	1	10.658	3.422	0.072	0.077
	MD-1	481.944	2	240.972	77.366	<.001	0.791
	PFD	22.214	1	22.214	7.132	0.011	0.148
	MD-1 * PFD	17.788	2	8.894	2.856	0.069	0.122
	Error	127.702	41	3.115			
	Total	3129.472	48				
	Corrected Total	681.88	47				
Thermal (Contralateral)	Corrected Model	410.877	6	68.479	13.637	<.001	0.666
	Intercept	3381.238	1	3381.238	673.337	<.001	0.943
	BW	5.594	1	5.594	1.114	0.297	0.026
	MD-1	336.72	2	168.36	33.527	<.001	0.621
	PFD	0.068	1	0.068	0.014	0.908	0
	MD-1 * PFD	0.919	2	0.459	0.091	0.913	0.004
	Error	205.886	41	5.022			
	Total	4024.143	48				
	Corrected Total	616.763	47				
Mechanical (Ipsilateral)	Corrected Model	96.994	6	16.166	65.436	<.001	0.905
	Intercept	367.227	1	367.227	1486.476	<.001	0.973
	BW	0.027	1	0.027	0.109	0.744	0.003
	MD-1	69.051	2	34.526	139.754	<.001	0.872
	PFD	8.695	1	8.695	35.198	<.001	0.462
	MD-1 * PFD	8.428	2	4.214	17.058	<.001	0.454
	Error	10.129	41	0.247			
	Total	482.211	48				
	Corrected Total	107.122	47				
Mechanical (Contralateral)	Corrected Model	67.359	6	11.227	15.798	<.001	0.698
	Intercept	517.257	1	517.257	727.874	<.001	0.947
	BW	1.272	1	1.272	1.79	0.188	0.042
	MD-1	65.028	2	32.514	45.753	<.001	0.691
	PFD	0.562	1	0.562	0.791	0.379	0.019
	MD-1 * PFD	0.261	2	0.13	0.184	0.833	0.009
	Error	29.136	41	0.711			
	Total	630.762	48				
	Corrected Total	96.495	47				

Table S7. ANCOVA analysis of the effects of MD-1 on ipsilateral and contralateral thermal and mechanical hypersensitivity, adjusted for body weight (BW) variations. Results show the source of variation, Type III Sum of Squares, degrees of freedom (df), Mean Square, F-value, significance level (Sig.), and Partial Eta Squared. Statistical significance is set at $P<0.05$.
